# Haplotype-resolved T2T genome and population resequencing provide insights into the domestication and mogroside biosynthesis of *Siraitia grosvenorii* (Cucurbitaceae)

**DOI:** 10.1093/hr/uhag103

**Published:** 2026-03-18

**Authors:** Yixuan Kou, Shulan Wang, Wei Xie, Li Dou, Dingguo Pan, Bowen Lai, Fuyan Mo, Panyu Yang, Dongchang Zeng, Sujuan Wei, Haimiao Wang, Zhiyong Zhang, Shaoqing Tang

**Affiliations:** Key Laboratory of Ecology of Rare and Endangered Species and Environmental Protection (Guangxi Normal University), Ministry of Education, Guilin 541006, Guangxi, China; Guangxi Key Laboratory of Landscape Resources Conservation and Sustainable Utilization in Lijiang River Basin, Guangxi Normal University, Guilin 541006, Guangxi, China; Guangxi University Engineering Research Center of Bioinformation and Genetic Improvement of Specialty Crops, Guangxi Normal University, Guilin 541006, Guangxi, China; Key Laboratory of Ecology of Rare and Endangered Species and Environmental Protection (Guangxi Normal University), Ministry of Education, Guilin 541006, Guangxi, China; Key Laboratory of Ecology of Rare and Endangered Species and Environmental Protection (Guangxi Normal University), Ministry of Education, Guilin 541006, Guangxi, China; Key Laboratory of Ecology of Rare and Endangered Species and Environmental Protection (Guangxi Normal University), Ministry of Education, Guilin 541006, Guangxi, China; Key Laboratory of Ecology of Rare and Endangered Species and Environmental Protection (Guangxi Normal University), Ministry of Education, Guilin 541006, Guangxi, China; Key Laboratory of Ecology of Rare and Endangered Species and Environmental Protection (Guangxi Normal University), Ministry of Education, Guilin 541006, Guangxi, China; Key Laboratory of Ecology of Rare and Endangered Species and Environmental Protection (Guangxi Normal University), Ministry of Education, Guilin 541006, Guangxi, China; Key Laboratory of Ecology of Rare and Endangered Species and Environmental Protection (Guangxi Normal University), Ministry of Education, Guilin 541006, Guangxi, China; Key Laboratory of Ecology of Rare and Endangered Species and Environmental Protection (Guangxi Normal University), Ministry of Education, Guilin 541006, Guangxi, China; Key Laboratory of Ecology of Rare and Endangered Species and Environmental Protection (Guangxi Normal University), Ministry of Education, Guilin 541006, Guangxi, China; Key Laboratory of Ecology of Rare and Endangered Species and Environmental Protection (Guangxi Normal University), Ministry of Education, Guilin 541006, Guangxi, China; Key Laboratory of Ecology of Rare and Endangered Species and Environmental Protection (Guangxi Normal University), Ministry of Education, Guilin 541006, Guangxi, China; Guangxi Key Laboratory of Landscape Resources Conservation and Sustainable Utilization in Lijiang River Basin, Guangxi Normal University, Guilin 541006, Guangxi, China; Guangxi University Engineering Research Center of Bioinformation and Genetic Improvement of Specialty Crops, Guangxi Normal University, Guilin 541006, Guangxi, China; Key Laboratory of Ecology of Rare and Endangered Species and Environmental Protection (Guangxi Normal University), Ministry of Education, Guilin 541006, Guangxi, China; Guangxi Key Laboratory of Landscape Resources Conservation and Sustainable Utilization in Lijiang River Basin, Guangxi Normal University, Guilin 541006, Guangxi, China; Guangxi University Engineering Research Center of Bioinformation and Genetic Improvement of Specialty Crops, Guangxi Normal University, Guilin 541006, Guangxi, China

## Abstract

Monk fruit (*Siraitia grosvenorii*, Cucurbitaceae) is globally renowned for its triterpenoid glycoside mogroside V, a high-intensity, non-caloric natural sweetener. However, its domestication and mogroside biosynthesis remain largely unknown. Here, we report a haplotype-resolved telomere-to-telomere (T2T) gapless genome for monk fruit, consisting of 14 chromosomes with genome sizes of 316.21 Mb (Hap1) and 316.07 Mb (Hap2). Comparative genomic analyses of the haplotypes revealed that structural variations and transposable elements have significantly contributed to genomic variation and architecture in monk fruit. Population genomic analyses based on 173 re-sequenced genomes indicated that cultivated monk fruit was mainly domesticated *in situ* from local wild populations in northern Guangxi of China, and that it likely experienced a mild domestication bottleneck, while exhibiting low genetic diversity. Demographic inference further revealed that the low genetic diversity is largely attributed to demographic changes driven by historical climate shifts. Selective sweeps were identified across all chromosomes of cultivated monk fruit, among which are genes exhibiting diverse putative functions and involved in various biosynthetic processes and secondary metabolism. This pattern of selective sweeps demonstrates the joint role of artificial selection and demographic changes in shaping the genomic landscape of cultivated monk fruit. Furthermore, comparative transcriptome analyses showed a pronounced temporally specific expression pattern among mogroside biosynthesis genes during fruit development and delineated additional candidate genes potentially involved in mogroside biosynthesis. This study not only provides insights into the domestication and mogroside biosynthesis of monk fruit but also lays a valuable genomic foundation for its molecular breeding and mogroside-targeted synthetic biology.

## Introduction

**Figure 1:**
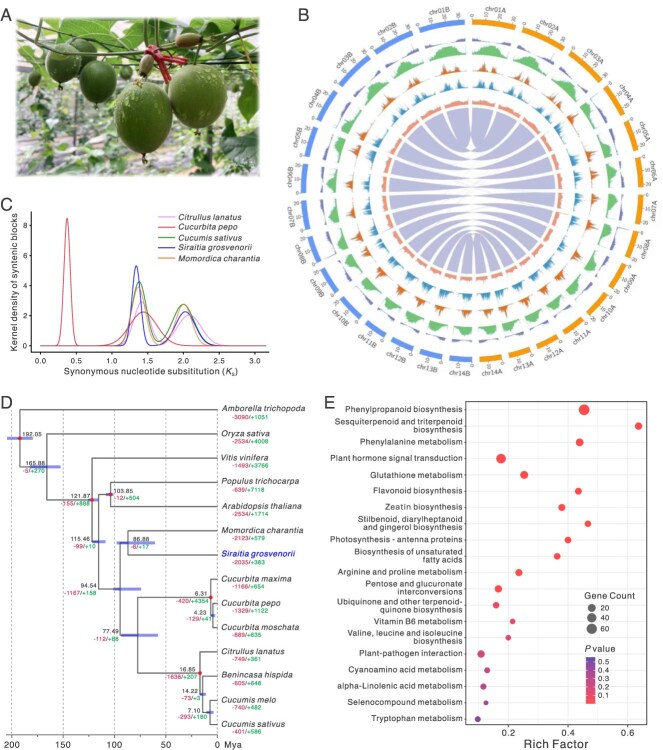
Haplotype-resolved T2T genome assembly and genome evolution of monk fruit. (A) The fruit of monk fruit ‘Qingpiguo.’ (B) Genomic features of the haplotype-resolved genome. Circles represent, from outermost to innermost, chromosome size (Mb), gene density, repeat density, *Gypsy* density, *Copia* density, GC content, and synteny between the two haplotypes. (C) Whole genome duplication (WGD) estimated through the distribution of synonymous nucleotide substitution (*K*_S_). (D) Inferred evolutionary relationship, divergence time, and gene family expansions and contractions. The red points represent the calibration times. (E) KEGG pathway enrichment of the expanded gene families.

The family Cucurbitaceae contains many of the world's most valuable plants, recognized for their edible and medicinal fruits, such as watermelon, cucumber, wax gourd, snake gourd, and bitter gourd [1]. *Siraitia grosvenorii* (2*n* = 2*x* = 28) [2], also known as monk fruit (Fig. 1A), is a member of the Cucurbitaceae family and has gained global recognition due to the triterpenoid glycoside mogroside V, which is commonly used as a natural sweetener with ~300 times sweeter than sucrose and no caloric content [3–5]. In addition, as a traditional Chinese medicinal herb, extracts of monk fruit exhibit various pharmacological effects, such as antioxidant, anti-inflammatory, immunomodulatory, and anti-tussive and expectorant properties [6–8].

Although monk fruit has been utilized for 2000 years as recorded in ancient texts [7, 9], its cultivation history in southern China is relatively short, spanning only ~300 years [2]. Currently, two cultivars, ‘Qingpiguo’ and ‘Hongmaoguo’, along with their derivatives, dominate cultivation due to their high productivity, accounting for over 98% of total yield [10]. Their cultivation is localized, primarily confined to several counties in Guilin of Guangxi (e.g. Yongfu, Lingui, and Longsheng). This restricted planting range stems largely from the fact that these cultivars were likely mainly domesticated *in situ* from local wild populations in Guilin [10, 11]. Moreover, the domestication process may have been accelerated by monk fruit's combined sexual and clonal reproductive traits: beneficial genetic variations arising from sexual reproduction can be rapidly fixed via clonal propagation [11, 12]. These multifaceted clues collectively suggest that cultivated monk fruit likely experienced relatively weaker domestication pressure and, thus, may have retained genomic genetic diversity comparable to that of wild populations, similar to domesticated grapes [13] and macadamia [14].

However, relatively low genetic diversity has been observed in cultivated populations (CULTs) of monk fruit compared with wild populations, based on RAPD and AFLP markers [10] as well as nuclear loci (*CHS* and *EDL2*) [11]. Phylogeographic analysis suggested that wild populations likely underwent demographic decline caused by historical climate fluctuations (e.g. Quaternary glaciations) [11]. Given that current cultivars were likely domesticated *in situ* relatively recently, the low genetic diversity may also inevitably have been shaped by such demographic changes. It remains unclear, however, whether the low genetic diversity in CULTs is primarily attributable to the domestication bottleneck and/or the demographic bottleneck. If the former, wild populations would be expected to harbor more abundant genomic variation than cultivated ones; if the latter, CULTs would likely have experienced a recent demographic decline alongside wild populations. To date, this issue has not been explored due to the scarcity of whole-genome sequence data for both cultivated and wild monk fruit.

The biosynthesis pathways of mogroside in monk fruit have been elucidated through the integration of genomics, transcriptomics, and metabolomics [3, 15, 16]. However, only two scaffold-level genomes of monk fruit have been reported to date, with significant discrepancies in their assembled sizes (~420 versus ~470 Mb). This inconsistency likely stems from high genome heterozygosity and technical limitations of the sequencing technologies used [3, 16]. With recent advances in ultra-long-read (ONT) and long-read (HiFi) sequencing, haplotype-resolved and gapless telomere-to-telomere (T2T) genomes have typically been assembled [17–19], enabling precise characterization of genomic variations and biosynthesis pathways in numerous horticultural crops [20–24]. Therefore, a high-quality T2T genome of monk fruit is vital to further improve our understanding of mogroside biosynthesis, particularly the temporal expression patterns of mogroside biosynthesis genes during fruit development. This will significantly enhance efforts to genetically regulate mogroside production.

Here, we present a haplotype-resolved T2T genome assembly of the monk fruit ‘Qingpiguo’, generated by integrating ultra-long-read (ONT), long-read (HiFi), and Hi-C sequencing. Genomic variation and evolution were characterized through comparative genomic analyses. Population genomic analyses based on 173 re-sequenced genomes further revealed the population structure, demographic history, and domestication of monk fruit. Additionally, the temporal expression patterns of mogroside biosynthesis genes across different fruit development stages were dissected in detail using comparative transcriptome analyses. These findings not only enrich the genomic repertoire available for Cucurbitaceae species but also enhance our understanding of genomic variation of cultivated and wild monk fruit. Our study establishes a valuable genomic foundation for genetic improvement and molecular breeding of monk fruit.

## Results

### Haplotype-resolved T2T genome assembly

A phased and gapless T2T genome was assembled for monk fruit ‘Qingpiguo’ in the present study. The genome size was assessed to be ~386 Mb with a high heterozygosity of ~1.39% by *K*-mer analysis based on 52.14 Gb (~139×) Illumina short reads (Fig. S1). Two genomic haplotypes with total lengths of ~335 and ~326 Mb were initially assembled based on 31.20 Gb PacBio HiFi reads (~91×) integrating 43.46 Gb ONT reads (~131×) and 48.82 Gb Hi-C reads (~111×). The two haplotypes exhibited contig N50 of 21.87 and 21.65 Mb, with maximum contig of 33.84 and 34.15 Mb, respectively (Table S1). The contigs of each haplotype assembly were then anchored on 14 chromosomes based on Hi-C reads with coverage rates of 94.08% and 96.73%, respectively (Fig. S2). After gap-closing based on ONT assembly and polishing based on HiFi reads, a final haplotype-resolved and gapless T2T genome was obtained for monk fruit with total lengths of 316.21 Mb for haplotype 1 (Hap1) and 316.07 Mb for Hap2 (Fig. 1B; Table S1).

Genome completeness evaluated using BUSCO showed that 96.35% and 96.82% of the orthologous genes were separately identified in the two genomic haplotypes. The quality value (QV) based on the *K*-mer statistics of HiFi reads and Illumina reads was 52.89 (corresponding to Hamming errors of 5.09 × 10^−6^) for Hap1 and 52.39 (5.77 × 10^−6^) for Hap2; the switch errors between the two haplotypes was 0.0013, indicating the high accuracy of the genome assembly. Mapping of sequencing reads showed that almost all reads were successfully mapped to the two genomic haplotypes (100% of Illumina reads, 99.94% of HiFi reads, and 99.98% of ONT reads). All 28 telomeres for each genomic haplotype were identified, and 14 centromeres for each genomic haplotype were predicted, with lengths ranging from 0.29 to 8.93 Mb (Table S2).

Totals of 21 853 protein-coding genes for Hap1 and 21 637 genes for Hap2 were predicted. The high completeness of gene sets was showed by BUSCO evaluation with 98.62% for Hap1 and 98.67% for Hap2. Of these genes, 95.26% in Hap1 and 95.60% in Hap2 were functionally annotated by at least one database from NR, KO, Swiss-Prot, GO, and Pfam. In addition, a comparable fraction of repetitive sequences was identified in both genomic haplotypes, 53.14% in Hap1 and 53.09% in Hap2. Of these repetitive sequences, transposable elements are the major contributor, including ~45% of LTR retroelements and ~11% of DNA transposons (Table S3).

### Comparative and evolutionary genomics

The *K*_S_ distribution analysis revealed that two common WGD events occurred in monk fruit compared with other Cucurbitaceae plants (Fig. 1C; Fig. S3), which are separately corresponding to core eudicot-common hexaploidy and cucurbit-common tetraploidy [25, 26]. However, no signature of the core eudicot-common hexaploidy was detected in *Cucurbita pepo* (Fig. 1C); this absence might be ascribed to the selective pressure after its recent exclusive WGD event [27, 28].

The evolutionary relationships of monk fruit were reconstructed by integrating 13 related species genomes (see section Materials and methods). A total of 349 single-copy orthologs were detected across all related species and used for inferring phylogenetic relationship and divergence time. The results exhibited that *S. grosvenorii* and *Momordica charantia* share a recent common ancestor, and the divergence time was inferred to 86.88 million years ago (Ma) with a confidence interval of 60.98 to 97.78 Ma (Fig. 1D). This time estimate is earlier than that in Cucurbitaceae phylogenies (~40–70 Ma [1]), possibly due to a scarcity of included species with assembled genomes within Cucurbitaceae.

The gene expansion and contraction were inferred based on 26 940 gene families identified across *S. grosvenorii* and 13 related species genomes. Of these gene families, 383 were expanded and 2035 were contracted (Fig. 1D). The expanded families were significantly enriched in 12 KEGG pathways (*P* < 0.05), such as sesquiterpenoid and triterpenoid biosynthesis, phenylpropanoid biosynthesis, plant hormone signal transduction, and flavonoid biosynthesis (Fig. 1E; Table S4).

**Figure 2:**
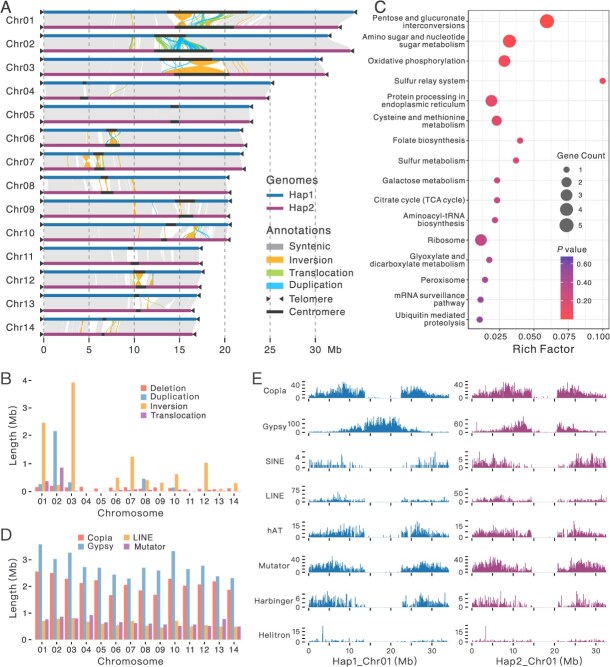
Genomic variations in the haplotype-resolved genome of monk fruit. (A) Visualization of structural variations on chromosomes, including inversion, translocation, duplication, and deletion/insertion. (B) Cumulative lengths of different types of structural variations across all chromosomes. (C) KEGG pathway enrichment of genes in specific chromosomal segments in Hap1. (D) Cumulative lengths of four major types of transposable elements (TEs) across all chromosomes. (E) Distributions of different types of TEs on chromosomes.

Structural variations (SVs), comprising 86 inversions, 252 translocations, 587 duplications, 3803 deletions, were discovered in Hap1 relative to Hap2 (Fig. 2A). The total length of SVs was 17.80 Mb, with the cumulative length of each SV type varying unevenly across all chromosomes and inversions accounting for the largest proportion (Fig. 2B). These inversions were confirmed by inspecting the breakpoints of HiFi reads mapped to the reference genome (Hap1) (Fig. S4). Notably, these SVs exhibited an overlap with 33.40% of the top 5% *F*_ST_ outliers and 20.06% of *π* ratio outliers (see Potential selection signatures during domestication). Additionally, the total length of specific chromosomal segments was 26.97 Mb in Hap1 and 31.84 Mb in Hap2, containing 379 and 594 genes, respectively (Fig. 2A). These genes were significantly enriched in three KEGG pathways (*P* < 0.05), including amino sugar and nucleotide sugar metabolism, pentose and glucuronate interconversions, and oxidative phosphorylation (Fig. 2C; Table S5).

Although the cumulative length of each TE family was comparable across all chromosomes in the two genomic haplotypes (Fig. 2D; Table S3), their density and distribution differed distinctly among the homologous chromosomes of the two haplotypes (Fig. 2E; Fig. S5). For example, *Gypsy* elements accumulated in the centromeres of Hap1 but were distributed along the chromosome arms of Hap2 across all homologous chromosomes (Fig. 2E; Fig. S5), indicating that TE expansion may shape the genomic architecture of monk fruit.

### Population structure and population genomics

Whole-genome resequencing data were produced for 149 wild and 18 cultivated individuals of monk fruit, with a mean depth ranging from 23.16× to 62.99×, coverage from 88.77% to 97.49%, and mapping rate (properly paired) from 86.76% to 95.42% (Table S6). A total of 10 200 087 high-quality biallelic SNPs were identified after a strict filtering, and used for subsequent population structure and population genomic analyses.

**Figure 3:**
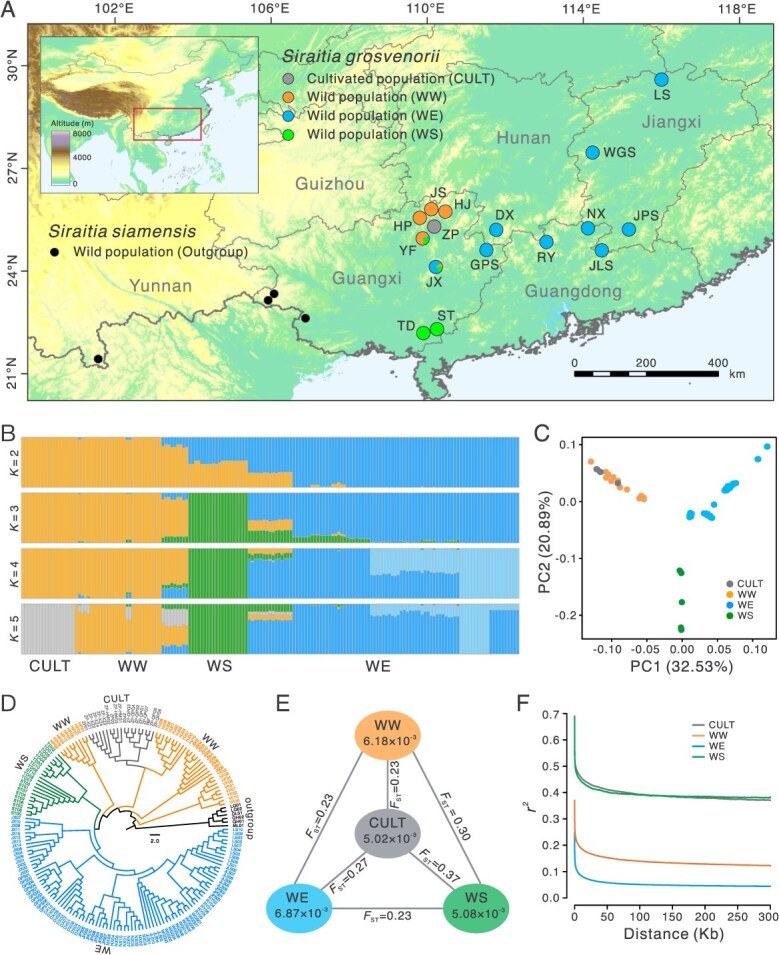
Population structure and population genomics of monk fruit. (A) Geographic distribution of sampled populations of monk fruit and its relative species *S. siamensis*. Base map downloaded from the Ministry of Natural Resources of China with an approval number GS(2016)1665 and GS(2019)1674. (B) Population structure at *K* (number of clusters) ranging from 2 to 5, revealing CULT and three genetic groups (WW, WS, and WE) within wild populations of monk fruit. (C) Principal component analysis (PCA) plot of the first two eigenvectors. (D) Phylogenetic tree inferred from whole-genome SNPs with outgroup *S. siamensis*. (E) Genome-wide nucleotide diversity (*π*) and population differentiation (*F*_ST_). (F) Linkage disequilibrium decay along physical distance measured by the squared correlation coefficients (*r*^2^).

Three geographically distinct genetic groups (WW, WS, and WE) were identified in wild monk fruit using Admixture: WW in northern Guangxi, WS in southern Guangxi, and WE in adjacent eastern areas (Fig. 3A and B). The three distinct groups were also confirmed by phylogenetic and PCA analyses (Fig. 3C and D; Fig. S6). In addition, CULT separated from group WW until *K* = 5 in admixture analysis (Fig. 3B), while it was entirely clustered within group WW in PCA and phylogenetic analyses (Fig. 3C and D; Fig. S6).

Genome-wide nucleotide diversity (*π*) was slightly lower in CULT (5.02 ± 3.07 × 10^−3^) than in WW (6.18 ± 3.23 × 10^−3^) and WE (6.87 ± 3.48 × 10^−3^), while comparable with WS (5.08 ± 2.82 × 10^−3^) (Fig. 3E; Table S7). Population differentiation (*F*_ST_) was higher between WW and WS (0.30 ± 0.10) than between WW and WE (0.23 ± 0.09) and between WE and WS (0.23 ± 0.10). Moreover, *F*_ST_ between CULT and WW (0.23 ± 0.12) was slightly lower than between CULT and WE (0.27 ± 0.10) and between CULT and WS (0.37 ± 0.12) (Fig. 3E; Table S7). In addition, a higher positive Tajima's *D* value was estimated in CULT (2.21 ± 1.09) than in WW (1.57 ± 1.01) and WE (1.65 ± 0.79), but slightly lower than in WS (2.48 ± 0.77) (Table S7).

Linkage disequilibrium (LD) value (*r*^2^) was higher in CULT (0.41 over a distance of 50 kb) than in WW (0.22) and WE (0.07) while comparable with that in WS (0.40) (Fig. 3F). However, the CULT did not exhibit slower LD decay relative to wild populations, with both showing an LD decay of ~200 bp when the *r*^2^ value drops to half of its maximum (Fig. 3F). These results imply that the CULT of monk fruit may have been exposed to relatively weaker selection pressure during domestication.

### Population demographic history

**Figure 4:**
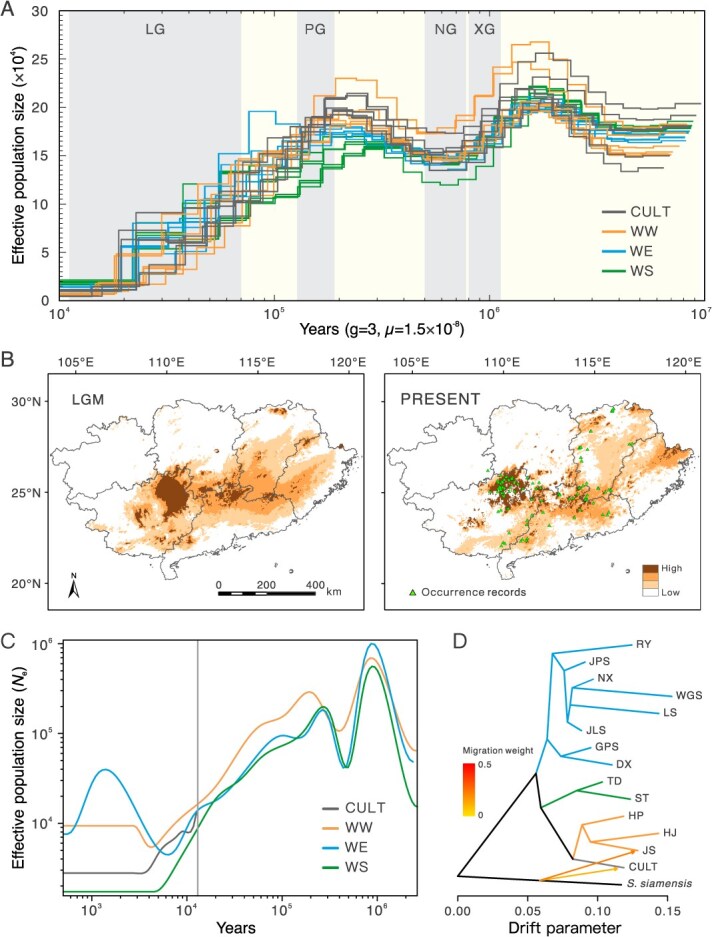
Population demographic history of monk fruit. (A) Historical changes of effective population size (*N*_e_) inferred using PSMC. XG, NG, PG, and LG represent the Xixiabangma Glaciation, Naynayxungla Glaciation, Penultimate Glacial, and Last Glacial, respectively, and are indicated by light gray bars. The Asian and Indian summer monsoons during interglacial are marked with light yellow bars. (B) Potential ecological niches during the Last Glacial Maximum (LGM) and the Present. Base map downloaded from the Ministry of Natural Resources of China with an approval number GS(2019)1674. (C) Historical changes of *N*_e_ and the split of the CULT from the wild population inferred using SMC++. (D) Population splits and mixture events inferred using TreeMix, with the optimum number of migration edges (*m* = 2).

The changes of effective population size (*N*_e_) inferred via PSMC model were almost concordant between CULT and three wild groups (WW, WS, and WE) with two waves of *N*_e_ increases (Fig. 4A). *N*_e_ increased initially to ~2.5 × 10^5^ individuals before 1.0 Ma, and then decreased to ~1.5 × 10^5^ individuals since the Xixiabangma Glaciation (~800–1170 thousand years ago, kya) to Naynayxungla Glaciation (~500–720 kya). Subsequently, *N*_e_ increased again to ~2.0 × 10^5^ individuals, and then decreased continuously since the Penultimate Glacial (~192–135 kya) and through the Last Glacial (~11–70 kya) [29, 30].

The potential species’ distributions were predicted using MaxEnt with a good model performance (AUC value >0.95) for four climatic periods, the Last Interglacial (LIG), the Last Glacial Maximum (LGM), the Mid-Holocene (MH), and the Present. The potential distributions of monk fruit have shown obvious changes since the LIG, with the change being particularly notable from the MH to the Present (Fig. 4B; Fig. S7). These results imply that, apart from historical climatic changes resulting from glacial–interglacial cycles, anthropogenic activities may also have impacted the distribution of monk fruit.

The demographic history of monk fruit over the past ~10 000 years was further revealed using SMC++ method. CULT split from wild group WW ~14 000 years ago, then underwent a continual reduction in *N*_e_ (Fig. 4C). Demographic changes across the three wild groups were asynchronous over the past ~10 000 years: the *N*_e_ of WE increased obviously and then decreased, WW experienced a slight increase in *N*_e_, while WS continuously decreased and then persisted at a small population size (Fig. 4C).

TreeMix analysis indicated that the CULT of monk fruit was split from the wild group WW (Fig. 4D; Fig. S8), aligning with the results of population structure analyses (Fig. 3). Moreover, the optimum number of migration edges (*m*) was estimated to be 2 and gene flow occurred from *Siraitia siamensis* (outgroup) to CULT and to wild population JS of monk fruit (Fig. 4D; Figs S8 and S9). The gene flow between them were also determined by ABBA–BABA analyses (Fig. S10).

### Potential selection signatures during domestication

**Figure 5:**
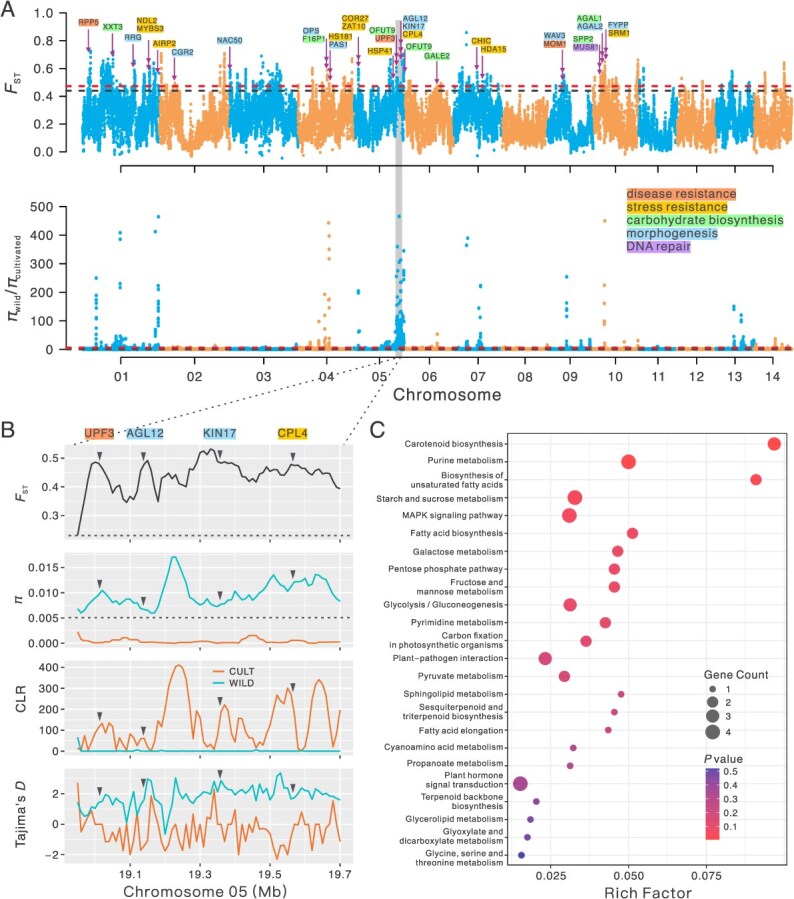
Potential selection signatures in the cultivated monk fruit genome during domestication. (A) Selective sweep across chromosomes identified using *F*_ST_ and *π* ratio (*π*_wild_*/π*_cultivated_), with candidate genes in these regions denoted. Black dashed lines indicate the top 5% thresholds for *F*_ST_ and *π* ratio values, and red lines represent the thresholds corrected following permutation tests. (B) Four candidate genes (*UPF3*, *AGL12*, *KIN17*, and *CPL4*) within regions on Chromosome 05 exhibiting positive selection signatures in *F*_ST_, *π*, CLR, and Tajima's *D* tests. Black triangles denote the chromosomal position of the four candidate genes. Dashed lines represent the genome-wide average *F*_ST_ between CULT and wild group (WW), and average *π* value in CULT. (C) KEGG pathway enrichment of genes located in selective sweep regions identified using *F*_ST_ and *π* ratio.

Selection signatures in genome of CULT of monk fruit were scanned based on *F*_ST_ and *π* ratio (*π*_wild_*/π*_cultivated_) between the CULT and the wild group (WW), and were further validated by Tajima’s *D* and CLR tests. After correction via permutation tests, selection signals were detected in 1717 genes by *F*_ST_ and 1138 genes by *π* ratio, with 331 genes shared between the two gene sets, which were annotated to be associated with disease resistance, stress resistance, carbohydrate biosynthesis, morphogenesis, DNA repair, and other molecular functions (Fig. 5A; Table S8). For example, four genes were identified within the selective sweep regions on Chromosome 05 (Fig. 5A), including *UPF3* (involved in related to defense responses to pathogenic infection), *AGL12* and *KIN17* (associated with plasnt morphogenesis), and *CPL4* (linked to abiotic stress resistance) (Table S8). These regions surrounding the four genes showed significantly higher *F*_ST_ than the whole-genome mean *F*_ST_ (0.23 ± 0.12), significantly reduced *π*, elevated CLR, and negative Tajima’s *D* in CULT comparing with wild population (Fig. 5B). The genes underlying the potential selective regions were further enriched in multiple KEGG pathways related to biosynthesis or secondary metabolism, including carotenoid biosynthesis, starch and sucrose metabolism, purine metabolism, fructose and mannose metabolism, fatty acid biosynthesis, and plant hormone signal transduction (Fig. 5C; Table S9).

### Expression of mogroside biosynthesis genes during fruit development

**Figure 6:**
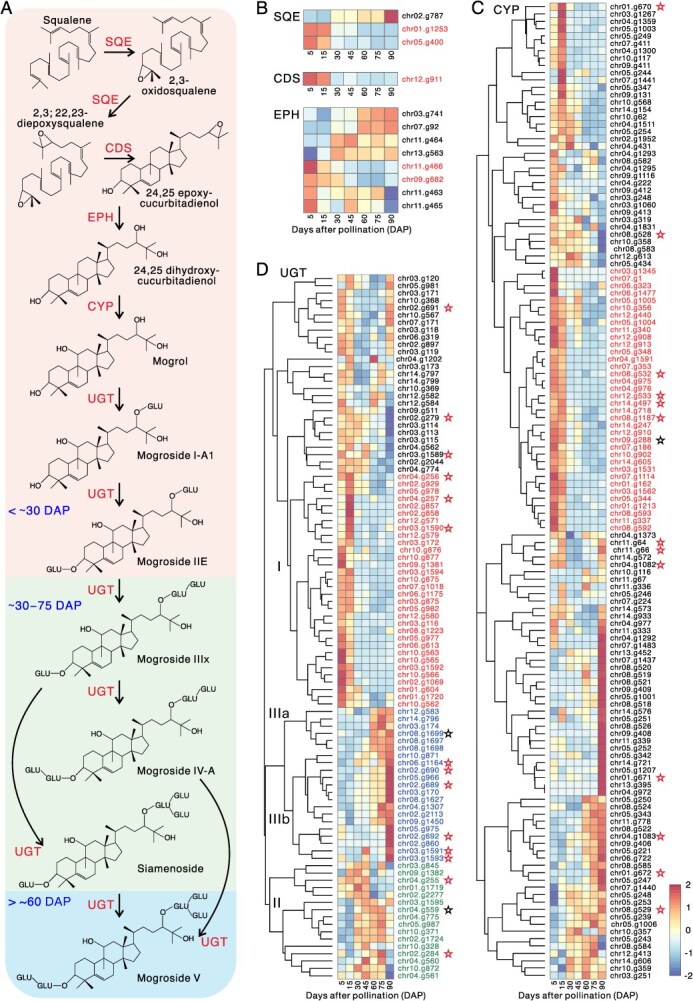
Expression patterns of mogroside biosynthesis genes during fruit development of monk fruit. (A) Mogroside biosynthesis pathway and its three phases divided according to mogroside metabolomic analysis [3]. (B) Expression of squalene epoxidase (*SQE*) genes, cucurbitadienol synthase (*CDS*) gene, and *EPH* genes. (C) Expression of *CYP* genes. (D) Expression of UDP-glucosyltransferase (*UGT*) genes. The genes labeled with black asterisks were confirmed to be involved in mogroside biosynthesis via functional expression assays [3], and those marked with red asterisks were exhibited through gene-concept network. The genes colored red, blue, and green are potentially involved in the three phases of the mogroside biosynthesis, respectively.

The expression patterns of mogroside biosynthesis genes were characterized in detail across seven key fruit development stages: 5, 15, 30, 45, 60, 75, and 90 days after pollination (DAP). Based on the metabolomic analysis of mogrosides during fruit development [3], mogroside biosynthesis in monk fruit is separated into three major phases (Fig. 6A): the first is the mogroside IIE biosynthesis phase, which is completed before ~30 DAP; the second is the mogroside IV-A and siamenoside biosynthesis phase, which occurs between ~30 and ~ 75 DAP; the third is the mogroside V biosynthesis phase, which takes place from ~60 DAP until full ripeness.

The significant phase-specific expression patterns were revealed among mogroside biosynthesis genes by comparative transcriptome analysis (Fig. 6B–D). During the first phase of mogroside biosynthesis (before ~30 DAP), mogrol undergoes initial glycosylation modifications catalyzed by UDP-glucosyltransferase (*UGT*) genes, generating mogroside IIE. This phase includes all gene families potentially involved in mogroside IIE biosynthesis, comprising two squalene epoxidase (*SQE*) genes, a cucurbitadienol synthase (*CDS*) gene, two epoxide hydrolase (*EPH*) genes, 36 cytochrome P450 (*CYP*) genes, and 32 *UGT* genes (subfamily I), all of which showed elevated expression (Fig. 6B–D). These genes formed a significant co-expression module (Fig. S11), indicating that a synergistic regulatory network between triterpene backbone synthesis and primary glycosylation is established early in fruit development. Subsequently, mogroside IV-A and siamenoside during the second phase (~30 to ~75 DAP), and mogroside V during the third phase (after ~60 DAP), are most likely catalyzed by *UGT* subfamily II (16 *UGT* genes) and subfamily III (21 *UGT* genes), respectively, with both subfamilies exhibiting increased expression (Fig. 6D). Furthermore, several candidate genes involved in mogroside biosynthesis were exhibited through gene-concept network, such as chr04.g256, chr02.g284, chr02.g690, and chr08.g532 (Fig. 6D; Fig. S12).

## Discussion

### Haplotype-resolved T2T genome assembly of monk fruit

A high-quality genome assembly is essential for investigating genomic variations and evolution in important economic crops. In the present study, we assembled a haplotype-resolved and gapless T2T genome composed of 14 chromosomes for monk fruit (Fig. 1B; Fig. S2). However, the size of the assembly is 316.21 Mb for Hap1 and 316.07 Mb for Hap2 (Fig. 1B; Table S1), which is significantly smaller than previous scaffold-level genome assemblies (~420 and ~470 Mb) [3, 16]. This discrepancy is likely attributable to two main factors: on the one hand, the complexity of the monk fruit genome, such as high heterozygosity (~1.39%; Fig. S1) and high fraction of repetitive sequences (~53.10%; Table S3); on the other hand, and primarily, the incomplete removal of redundant contigs resulting from technical limitations associated with sequencing and assembly approaches employed in the previous study (Fig. S13) [16]. Advances in genome sequencing and assembling technologies, particularly ultra-long-read (ONT) and Hi-C sequencing used in the present study, enable the precise assembly of haplotype-resolved T2T genomes [17–19], thus resolving the genome size estimate of monk fruit.

Although the two haplotypes of monk fruit genome exhibit high collinearity, there exist numerous SVs between them. We observed large segment inversions on several chromosomes, as well as deletions/insertions and specific chromosomal segments on all chromosomes (Fig. 2A). These variations directly contribute to differences in chromosome length and gene number among the homologous chromosomes of the two haplotypes (e.g. *Vitis* accessions [31] and *Areca catechu* [32]). For instance, the total length of specific chromosomal segments is 26.97 Mb in Hap1 and 31.84 Mb in Hap2, and contain 379 and 594 genes, respectively (Fig. 2A). In addition, our genome assembly also revealed that the density and distribution of each transposable element (TE) family are similar across chromosomes within each genomic haplotype but distinct between the two haplotypes. For instance, *Gypsy* elements are concentrated in the centromeres of Hap1 but in the chromosome arms of Hap2 (Fig. 2E), indicating that TEs may contribute to shaping the genomic architecture of monk fruit (e.g. *Vitis vinifera* [33] and *Capsicum* species [34]). Therefore, the new haplotype-resolved T2T genome assembled in the present study significantly improves our understanding of genomic variation and architecture in monk fruit.

Following comparative genomic analyses, two shared WGD events, the core eudicot-common hexaploidy and cucurbit-common tetraploidy, which have been reported in other Cucurbitaceae species [25, 26], were also identified in the monk fruit genome (Fig. 1C). These WGD events potentially drove the expansion of gene families and thereby shaped the specialized genomic functions of monk fruit. For instance, the expanded gene families are enriched in pathways including sesquiterpenoid and triterpenoid biosynthesis, plant hormone signal transduction, phenylpropanoid biosynthesis, and flavonoid biosynthesis (Fig. 1D), which may play crucial roles in plant development and responses to diverse stresses [35–38].

### Mild domestication bottleneck in monk fruit

Current cultivars of monk fruit may have been mainly domesticated from local wild populations in northern Guangxi. Specifically, the CULT consistently clustered within genetic group WW of wild monk fruit distributed in northern Guangxi (Fig. 3), and the population differentiation (*F*_ST_) between CULT and WW (0.23 ± 0.12) was slightly lower than that between CULT and the other two genetic groups of wild monk fruit (WE: 0.27 ± 0.10; WS: 0.37 ± 0.12) (Fig. 3E). This result aligns with the findings of previous phylogeographic analyses of monk fruit [11].

However, unlike most cereal crops, such as barley, rice, bread wheat, and maize [39–41], cultivated monk fruit likely experienced a mild domestication bottleneck. First, the genomic genetic diversity (*π*) in CULT (5.02 × 10^−3^) was merely slightly lower than that in wild populations (5.08–6.18 × 10^−3^), and the LD decay rate in CULT was almost identical to that in wild populations (Fig. 3F). In contrast, crops that underwent intense domestication bottlenecks typically exhibit substantial reductions in genetic diversity and marked elevation in LD [42, 43]. Second, the domestication history of cultivated monk fruit may be relatively short. Its cultivation in southern China has been documented for only ~300 years [2], which is far shorter than the onset of human-associated plant domestication in China (~10 000 years ago) [42]. However, this seems to contradict the time estimated through demographic inference: the CULT split from wild group WW ~14 000 years ago (Fig. 4C). This is likely largely because current cultivars of monk fruit are just entering the second stage of domestication: only a tiny minority of desirable alleles become gradually fixed through *in situ* selection of desirable germplasm [42]. Thus, the estimation is more likely to reflect the time of population substructure in wild monk fruit, rather than the origin time of cultivated monk fruit, though additional evidence is required to clarify this issue. Third, monk fruit can produce sexual offspring via pepos, and subsequently undergo clonal propagation through developed fleshy tuberous roots and stem nodes [12]. Desired phenotypic traits can be quickly fixed through clonal propagation of stem cuttings, and concurrently, a large majority of genomic variations from wild populations can be preserved in the current cultivars. This breeding strategy potentially weakens the domestication bottleneck of monk fruit, which is also observed in other clonally propagated crops, such as grapes [13] and macadamia [14].

In fact, a strong population bottleneck was detected in cultivated monk fruit through demographic inference. However, this bottleneck cannot be entirely ascribed to domestication, as the CULT shares similar demographic changes with wild populations (Fig. 4A). Specifically, changes in effective population size (*N*_e_) largely coincided with historical climate events: two waves of *N*_e_ increases occurring before 1.0 and 0.2 Ma (Fig. 4A) corresponded to the strengthening of the Indian and Asian summer monsoons during the interglacial periods [44, 45]; whereas a continuous decrease in *N*_e_ after 0.2 Ma was associated with the Last Glacial and the Penultimate Glacial (Fig. 4A and B) [29, 30], resulting in extremely small population sizes persisting after ~10 000 years ago, particularly in the CULT (~2.8 × 10^3^ individuals) and the wild group WS (~1.5 × 10^3^ individuals) (Fig. 4A and C). Although the *N*_e_ of the wild groups WW and WE recovered slightly after ~5000 years ago, this recovery might be due to gene flow from the related species *S. siamensis* to group WW (Fig. 4D) and population substructure in the widely distributed group WE (Fig. 3A and B). Therefore, the strong population bottleneck in cultivated monk fruit may largely be due to demographic changes driven by historical climate shifts, rather than domestication.

It is likely that precisely due to the mild domestication bottleneck and strong demographic bottleneck, an abundance of selective sweeps has been detected across all chromosomes of cultivated monk fruit (Fig. 5A). The genes underlying these selective sweeps exhibit diverse putative functions (Fig. 5A) and are involved in various biosynthetic processes and secondary metabolism (Fig. 5C; Table S9), indicating that multiple demographic and selective processes have most likely shaped functional gene variations in cultivated monk fruit. For example, genetic accessions carrying plant disease defense-related genes *RPP5*, *UPF3* and *MOM1* [46–48], identified within the selective sweep regions in the monk fruit genome (Fig. 5A), are favored not only by artificial selection but also by natural selection, despite their tendency to be lost during domestication [49, 50]. Additionally, 33.40% of *F*_ST_ outliers and 20.06% of *π* ratio outliers overlapped with SVs in the monk fruit genome, respectively (Figs 2A and 5A), indicating that SVs also potentially contributed gene variations in the demographic and selective processes (e.g. grapes [23, 31, 51, 52] and *Oryza sativa* [53]). Nonetheless, determining what of role of SVs and the genes associated with them during domestication will require further investigation, especially genome-wide association analysis of agronomic traits in cultivated monk fruit.

### Additional candidate genes potentially involved in mogroside biosynthesis

Combined with the metabolomic analysis of mogrosides during fruit development [3], comparative transcriptome analysis in the present study revealed a pronounced temporally specific expression pattern among mogroside biosynthesis genes, and divided mogroside biosynthesis into three major phases (Fig. 6A): the first is the mogroside IIE biosynthesis phase (before ~30 DAP); the second is the mogroside IV-A and siamenoside biosynthesis phase (~30 to ~75 DAP); and the third is the mogroside V biosynthesis phase (from ~60 DAP until full ripeness). In the previous study, two primary *UGT* genes (chr04.g559 and chr08.g1699) or the gene subfamilies (II and IIIa) to which they belong were confirmed to be involved in mogroside biosynthesis via functional expression assays (Fig. 6D) [3]. However, a novel *UGT* gene subfamily (I) was found to exhibit significant expression during the first phase (before ~30 DAP) and to co-express with the upstream genes (*SQE*, *CDS*, *EPH*, and *CYP*) involved in mogroside biosynthesis, such as candidate genes chr04.g256, chr04.g257, and chr03.g1590 (Fig. 6; Fig. S12). This indicates that the subfamily is likely primarily involved in mogroside biosynthesis from mogrol to mogroside IIE. In contrast to the previous study [3], *UGT* gene subfamily II (chr04.g559) did not show significant expression during the first phase (Fig. 6D); instead, its expression peaked predominantly during the second phase (~30 to ~75 DAP). This suggests that the subfamily is likely primarily involved in the biosynthesis of mogroside IV-A and siamenoside rather than that of mogroside IIE, such as candidate genes chr04.g255 and chr02.g284 (Fig. 6D; Fig. S12).

Analogously, the *UGT* gene subfamily IIIa (chr08.g1699) was reported to be involved in mogroside biosynthesis from mogroside IIE to mogroside V [3], a process corresponding to the second and third phases of mogroside biosynthesis as defined in the present study (Fig. 6A). However, our results demonstrated that this subfamily exhibits elevated expression exclusively during the third phase (from ~60 DAP until full ripeness), potentially contributing to the synthesis of mogroside V from mogroside IV-A and siamenoside (Fig. 6D). Notably, further findings from *in vitro* glucosylation assays revealed that chr08.g1699 exhibits relatively low efficiency in glycosylating mogroside IV-A to mogroside V [4]. We therefore postulate that subfamily IIIa (chr08.g1699) likely acts synergistically with another subfamily, IIIb, which shows significantly high expression at ~90 DAP, such as candidate genes chr02.g690, chr02.g692, and chr06.g1164 (Fig. 6D; Fig. S12). Importantly, this high expression of subfamily IIIb coincides precisely with the dramatic accumulation of mogroside V observed during the later phases of fruit development [3].

Overall, additional candidate genes potentially involved in mogroside biosynthesis were identified in the present study through the dissection of their temporal expression patterns. Although the functions of these genes require further validation, these results lay an important foundation for future research on gene editing and mogroside synthetic biology related to mogroside biosynthesis. Furthermore, they provide direct guidance for the agricultural production of monk fruit. For instance, the optimal harvest time can be determined based on specific needs: as the fruit hardly enlarges further after ~30 DAP (Fig. S14), harvesting at ~75 DAP will not compromise yield and will maximize the siamenoside content in the fruit [3].

## Materials and methods

### Plant materials and sequencing

The popular cultivar ‘Qingpiguo’ of monk fruit, cultivated in Guilin of China, was used for *de novo* genome assembly. Young leaves were sampled for genomic DNA extraction and short-read and long-read sequencing; young leaves, flowers, stem, root, and fruits at 45 days after control pollination were sampled for total RNA extraction and third-generation full-length transcriptome (long-read) sequencing. A total of 173 individuals were sampled for whole-genome (short-read) resequencing, including 18 from cultivars, 149 from 15 wild populations across their entire geographical distribution of *S. grosvenorii*, and 6 individuals of *S. siamensis* from four populations used as an outgroup (Table S10). Fruit pulp samples of the cultivar ‘Qingpiguo’ at seven development stages, 5, 15, 30, 45, 60, 75, and 90 DAPss, were collected for transcriptome (short-read) sequencing, with three biological replicates for each stage. All collected samples were promptly frozen in liquid nitrogen and subsequently preserved at −80°C for DNA and RNA extraction.

Genomic DNA was retrieved from plant tissue using the DNeasy Plant Mini Kit (QIAGEN). DNA quality was checked using an agarose gel electrophoresis apparatus (Liuyi Biotechnology) and the NGS™ dsDNA HS Assay Kit on the Qubit 4.0 Fluorometer (Invitrogen). Total RNA was obtained with TRIzol RNA extraction reagent (Thermo Fisher). RNA quality was checked using an agarose gel electrophoresis apparatus (Liuyi Biotechnology) and the RNA 6000 Nano Kit on the Bioanalyzer 2100 system (Agilent). After quality assessment, genomic DNA and total RNA were served for library construction, followed by long-read and short-read sequencing.

In long-read sequencing, 20 kb libraries for genomic DNA and total RNA were prepared by SMRTbell™ Template Prep Kit 1.0 and sequenced on a PacBio Revio platform (Pacific Biosciences) to generate HiFi reads. The ultra-long Nanopore library (>50 kb) for genomic DNA was processed using the Ligation Sequencing SQK-LSK109 Kit and sequenced on a PromethION sequencer (Oxford Nanopore Technologies) to generate ONT reads. The size of target library for long-read sequencing was picked via a BluePippin Recovery System (Sage Science).

In short-read sequencing, 150 bp paired-end libraries were constructed for genomic DNA and total RNA using TruSeq DNA PCR-free prep kit. The high-throughput chromosome conformation capture (Hi-C) library was constructed for genomic DNA through the following process: chromatin crosslinking in 1% formaldehyde, chromatin digestion with the restriction enzyme dpnII, marking of DNA ends with biotin, and shearing to 200–400 bp after DNA ligation and purification. These libraries were then sequenced using an Illumina NovaSeq platform (Illumina) to produce 2 × 150 bp paired-end reads.

### Genome assembly

Before assembling the genome of monk fruit, genome size, repetitive fraction, and heterozygosity were evaluated using Jellyfish v2.3.0 (*K*-mer = 19) [54] and GenomeScope v2.0 [55], based on Illumina short reads.

Two draft genomic haplotypes were constructed by Hifiasm v0.24.0 [18], based on HiFi reads integrating Hi-C and ONT reads. The valid interaction pairs of Hi-C reads were retained after twice alignment with draft genome using Hi-C-Pro v3.1.0 [56], and then were used to connect contigs into chromosome-level genomic haplotypes using 3D-DNA v201008 [57]. Misassembled contigs were inspected and corrected manually using Juicebox v1.11.08 [58]. Subsequently, the preliminary assembly of ONT reads was executed using nextDenovo v2.5.0 [59] and applied to fill gaps on the two genomic haplotypes using TGS-GapCloser v1.1.1 [60]. The ONT reads, aligned to the ends of the above genomic haplotypes using Winnowmap v2.03 [61], were further used to assembly the sequence of telomeres using medaka_consensu v1.7.3 (Oxford Nanopore Technologies). Finally, the haplotype-resolved T2T genome was obtained following polishing with HiFi reads using nextpolish2 [62].

Genome continuity was determined through counting gaps on the assembly, while genome completeness was surveyed using BUSCO v5.4.5 (eudicots_obd10) [63]. The QV and Hamming errors for the genome assembly was computed with Merqury v1.3 [64], based on the *K*-mer statistics from Illumina reads and HiFi reads. Genome phasing accuracy was evaluated by calculating switch errors between haplotypes using a public pipeline (https://github.com/tangerzhang/calc_switchErr). In addition, telomere and centromere sequences were identified and predicted by tidk v0.2.0 [65] with the telomeric repeat motif ‘AAACCT’ or ‘AGGGTTT’ and CentroMiner tool in quartet v1.2.4 [66], respectively.

### Genome annotation

Transposable elements (TEs) were annotated by homology search strategies and *ab initio* prediction. In *ab initio* prediction, TE boundaries were identified and family relationships were classified by RECON v1.0.8 [67] and RepeatScout v1.0.6 [68] in software RepeatModeler v2.0.4 [69]. In homology search, the genome of monk fruit ‘Qingpiguo’ was searched against the Repbase database (version: 20150807) [70] by RepeatMasker v4.1.4 [71].

The structures of coding genes were predicted by three strategies, transcriptome-assisted, homology-based, and *de novo* prediction. *de novo* prediction of gene model was performed by Augustus v3.3.2 [72], SNAP v20170301 [73], GlimmerHMM v3.0.4 [74], and GeneMark v4.71 [75]. The protein sequences from *M. charantia*, *Cucumis sativus*, *Citrullus lanatus*, and *Cucurbita maxima* genomes (Table S11) were used for homology-based prediction using GeMoMa [76] and Exonerate v2.2.0 [77]. The full-length transcriptome data, after alignment to the assembled genome, was employed to assist gene prediction with PASA v2.5.2 [78]. Finally, all prediction results from the three strategies were combined into a consensus gene set via EvidenceModeler v20120625 [79]. The gene completeness was examined through BUSCO v5.4.5 (eudicots_obd10) [63].

The functions of coding genes were inferred via Diamond v2.0.14.152 [80] by searching their sequences against protein sequences in the Swiss-Prot database and NCBI non-redundant protein (NR) database with an 1e^−5^ E-value. The KEGG Automatic Annotation Server was employed to generate KEGG Ortholog (KO) and KEGG Pathway [81]. The protein domain based on Pfam database and gene ontology (GO) term were annotated using InterProScan v5.61-93.0 [82].

### Comparative genomic analysis

Whole-genome duplication (WGD) events were estimated using the distribution of synonymous nucleotide substitution (*K*_S_). The *K*_S_ values among homologous gene pairs was computed using WGDI v0.74 [83] for the genome of monk fruit and four related species, *M. charantia*, *C. sativus*, *C. lanatus*, and *C. pepo* (Table S11). A dot plot was also generated to visualize WGD signals for the genome of monk fruit by WGDI.

Protein sequences were retrieved from 13 related species’ genomes for ortholog analyses, including *M. charantia*, *C. sativus*, *C. maxima*, *C. pepo*, *C. lanatus*, *Cucumis melo*, *Cucurbita moschata*, *Benincasa hispida*, *Arabidopsis thaliana*, *Populus trichocarpa*, *V. vinifera*, *O. sativa*, and *Amborella trichopoda* (Table S11). OrthoFinder v2.5.5 [84] was employed to discovery gene families and single-copy orthologues. The single-copy genes were utilized to build the species evolutionary relationships via RAxML-NG v1.2.0 [85] with *A. trichopoda* as an outgroup. The MCMCtree application in PAML v4.10.7 [86] was employed to infer species divergence times, with fossil calibrations obtained from TimeTree (www.timetree.org). Expansion and contraction of gene families were detected using CAFE5 [87]. Genes from expanded families were subsequently subjected to analysis of KEGG pathway enrichment through R package clusterProfiler [88].

Two haplotypes of the monk fruit genome were aligned using Minimap2 v2.28 [89] with the default parameters, and then the syntenic regions and SVs (insertion/deletion, inversion, translocation, and duplication) were identified using SyRI v1.7.0 [90]. Plotsr v1.1.1 [91] was used to visualize these SVs and syntenic regions. To validate these SVs, the HiFi reads were aligned to the reference genome (Hap1) using Minimap2 v2.28 [89], and the breakpoints of SVs were manually inspected using IGV v2.7.2 [92]. The genes related to SVs were then subjected to analysis of KEGG pathway enrichment by R package clusterProfiler [88].

### Read mapping and variation calling

Raw Illumina reads of whole-genome resequencing were assessed by FastQC v0.12.1 (https://www.bioinformatics.babraham.ac.uk/projects/fastqc/), and then filtered via Trimmomatic v0.39 [93] to remove adapters and low-quality base (<20). The resulting clean reads were subsequently aligned to the Hap1 genome of monk fruit using BWA 0.7.18 [94] with BWA-MEM algorithm. Picard v2.25.5 (https://broadinstitute.github.io/picard/) was used to filter out duplicated reads. The resulting BAM file was then sorted and indexed with SAMtools v1.20 [95] for genomic variant discovery.

The HaplotypeCaller in GATK v4.4.0.0 (https://gatk.broadinstitute.org/) was used for scalable variant calling for each sample to generate an intermediate GVCF file. All of the GVCF files were then consolidated by GenomicsDBImport and used for joint variant discovery by GenotypeGVCFs. Genomic variant SNPs were filtered by VariantFiltration in GATK v4.4.0.0, following the criteria described in our previous study [53]: quality score (QUAL) > 40.0, variant quality (QD) > 2.0, and mapping quality (MQ) > 30.0. The resulting variants were further filtered using VCFtools v0.1.16 [96] with missing genotypes of <95% and minor allele frequency of >0.05 across all samples.

### Population structure and population genomic analysis

Population structure was inferred using a block relaxation algorithm in Admixture v1.3.0 [97], with the algorithm terminating when the delta log-likelihood fell below 0.0001 across iterations. To further explore the population structure, a principal component analysis (PCA) was executed through Plink v1.90 [98], and a phylogenetic tree was built with RAxML-NG v1.2.0 [85] under GTR + G model.

Population genomic statistics, including genome-wide nucleotide diversity (*π*), Tajima’s *D*, and fixation statistic (*F*_ST_) for population differentiation were computed by VCFtools v0.1.16 [96]. LD decay was quantified by the squared correlation coefficients (*r*^2^) between all SNP pairs using PopLDdecay v3.4.3 [99].

### Population demographic inference

The historical changes of effective population size (*N*_e_) of monk fruit were inferred using PSMC v0.6.5 [100] with -p ‘4 + 25*2 + 4 + 6’ and other default parameters based on whole-genome resequencing data. The population size histories and split time were further estimated using SMC++ v1.14.0 [101] with ‘estimate’ and ‘split’ commands. A universal mutation rate of 1.5 × 10^−8^ per site per generation for all dicots [28] and a generation time of 3 years observed in our field investigations over many years were used to scale the demographic history.

Population splits and mixture events were deduced using TreeMix v1.13 [102] with -m spanning from 0 to 10, -k 500, -bootstrap 100, and *S. siamensis* as root taxon. The optimum number of migration edges (*m*) was estimated using Δ*m* calculated by R package OptM [103]. ABBA–BABA tests were further performed to estimate gene flow among populations or groups using the Dtrios program in Dsuite [104].

### Ecological niche modeling

The historical climatic niches were simulated to further estimate the effect of climatic change on population genetic structure of monk fruit. A total of 119 valid occurrence records of monk fruit, sourced from the Chinese Virtual Herbarium (CVH, www.cvh.ac.cn) and the Global Biodiversity Information Facility (GBIF, www.gbif.org), were utilized for niche modeling. Nineteen bioclimatic variables across four climatic periods, the LIG, the LGM, the MH, and the Present, were retrieved from WorldClim database at a 2.5-minute spatial resolution (www.worldclim.org [105]). To reduce overfitting in niche models, nine bioclimatic variables (bio2, bio3, bio5, bio7, bio8, bio12, bio15, bio18, and bio19) were kept for subsequent niche modeling, as their pairwise Pearson correlation coefficients met the criterion of |*r*| ≤ 0.80.

The climatic niches of monk fruit were modeled with MaxEnt v3.4.3 [106], where 20% of species records were used for model testing and 80% for model training, with 10 replicates per run following our previous study [107]. Model accuracy was assessed via the area under the ROC curve (AUC value) [108]. The predictions of climatic niches were visualized in ArcGIS v10.2 (ESRI, Redlands, CA, USA).

### Selection signature identification

Genomic signatures of selection in CULT of monk fruit were identified using VCFtools v0.1.16 [96], based on population fixation statistic (*F*_ST_), nucleotide diversity (*π*) ratio (*π*_wild_*/π*_cultivated_), and Tajima’s *D*. *F*_ST_ and *π* ratios were calculated using a 50 kb sliding window with a 10 kb step size, while Tajima’s *D* using a 10-kb nonoverlap sliding window. The composite likelihood ratio (CLR) test was further employed to detect genomic signatures of selection utilizing SweeD v4.0.0 [109] with a 10-kb nonoverlap sliding window. The top 5% outliers on *F*_ST_ and *π* ratio distributions were corrected separately following permutation tests, and their overlapping outliers were defined as the final selective signals in CULT, which were further validated in CLR and Tajima's *D* tests. The genes underlying the outliers were then subjected to KEGG pathway enrichment by R package clusterProfiler [88].

### Transcriptome data processing

The raw RNA paired-end reads (~6 Gb) from each fruit pulp sample of monk fruit ‘Qingpiguo’ were filtered using Trimmomatic v0.39 [93] to remove adapters and low-quality base (<20). The resulting clean reads were then aligned to the Hap1 genome of monk fruit using HISAT2 v2.2.1 [110]. Subsequently, featureCounts v2.0.8 [111] was used to calculate the counts of aligned reads for genes in the mogroside biosynthesis pathway. Following count normalization, gene expression was quantified using DESeq2 v1.30.1 [112], with a significance threshold of adjusted *P* value <0.05 and |log₂(Fold Change)| ≥ 1. The clustered heatmap for these genes were produced using pheatmap v1.0.13 (https://github.com/raivokolde/pheatmap). Finally, gene-concept network between these genes and KEGG pathways was constructed using R package enrichplot (https://github.com/YuLab-SMU/enrichplot).

## Supplementary Material

Web_Material_uhag103

## Data Availability

All the raw sequencing data and the genome assembly from this project have been deposited in the National Genomics Data Center (NGDC, https://ngdc.cncb.ac.cn/) under BioProject number PRJCA045668.
