# Supplementary material for: Haplotype-resolved T2T genome and population resequencing provide insights into the domestication and mogroside biosynthesis of Siraitia grosvenorii (Cucurbitaceae)
Source: Hortic Res. 2026 Mar 18;13(7):uhag103. doi: 10.1093/hr/uhag103 (PMC13283849; doi:10.1093/hr/uhag103)
Supplement: Web_Material_uhag103 [file web_material_uhag103.zip › Supplementary files_r2.docx]

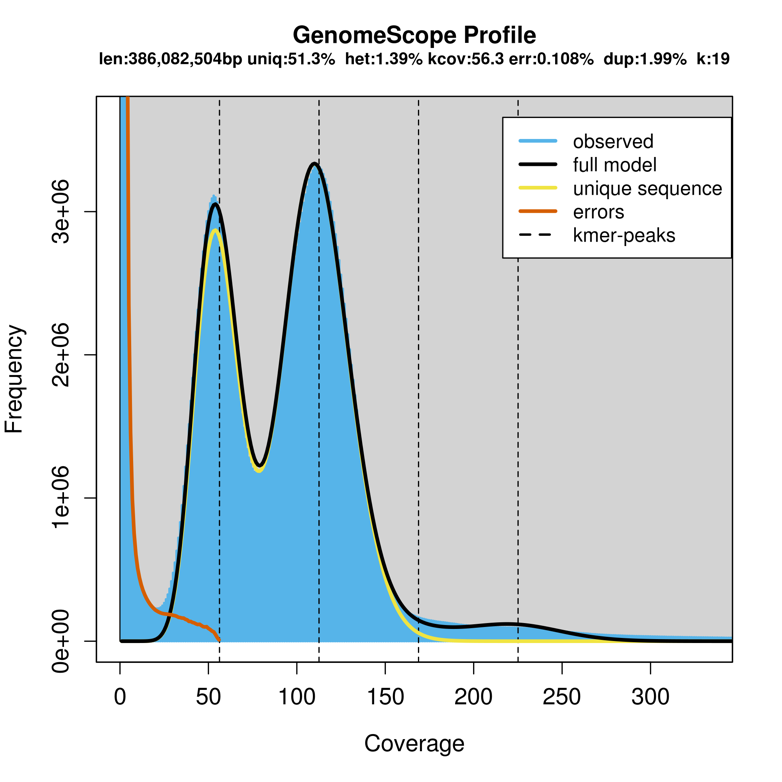


**Figure S1.** Evaluation of the genome size and complexity of monk fruit using the *K*-mer method based on 52.14 Gb (~135×) Illumina short reads.


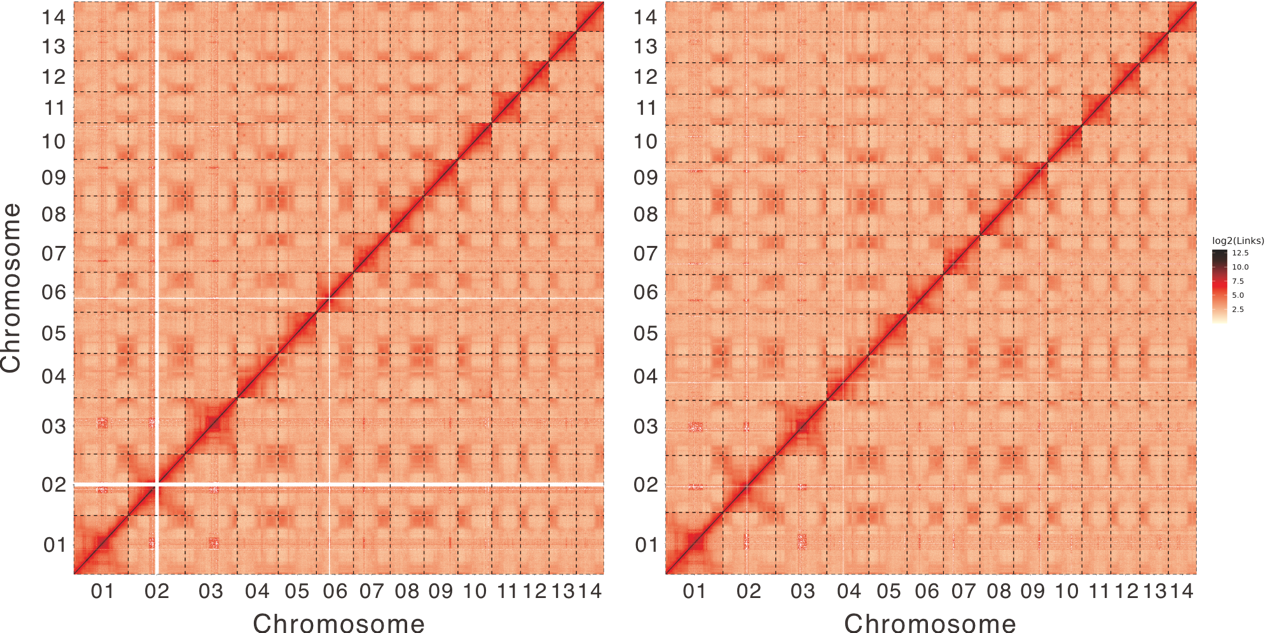


**Figure S2.** Genome-wide Hi-C interaction heatmaps of the 14 chromosomes for genomic hap1 (left) and hap2 (right) of monk fruit. The color in the heatmap represents the interaction intensity.


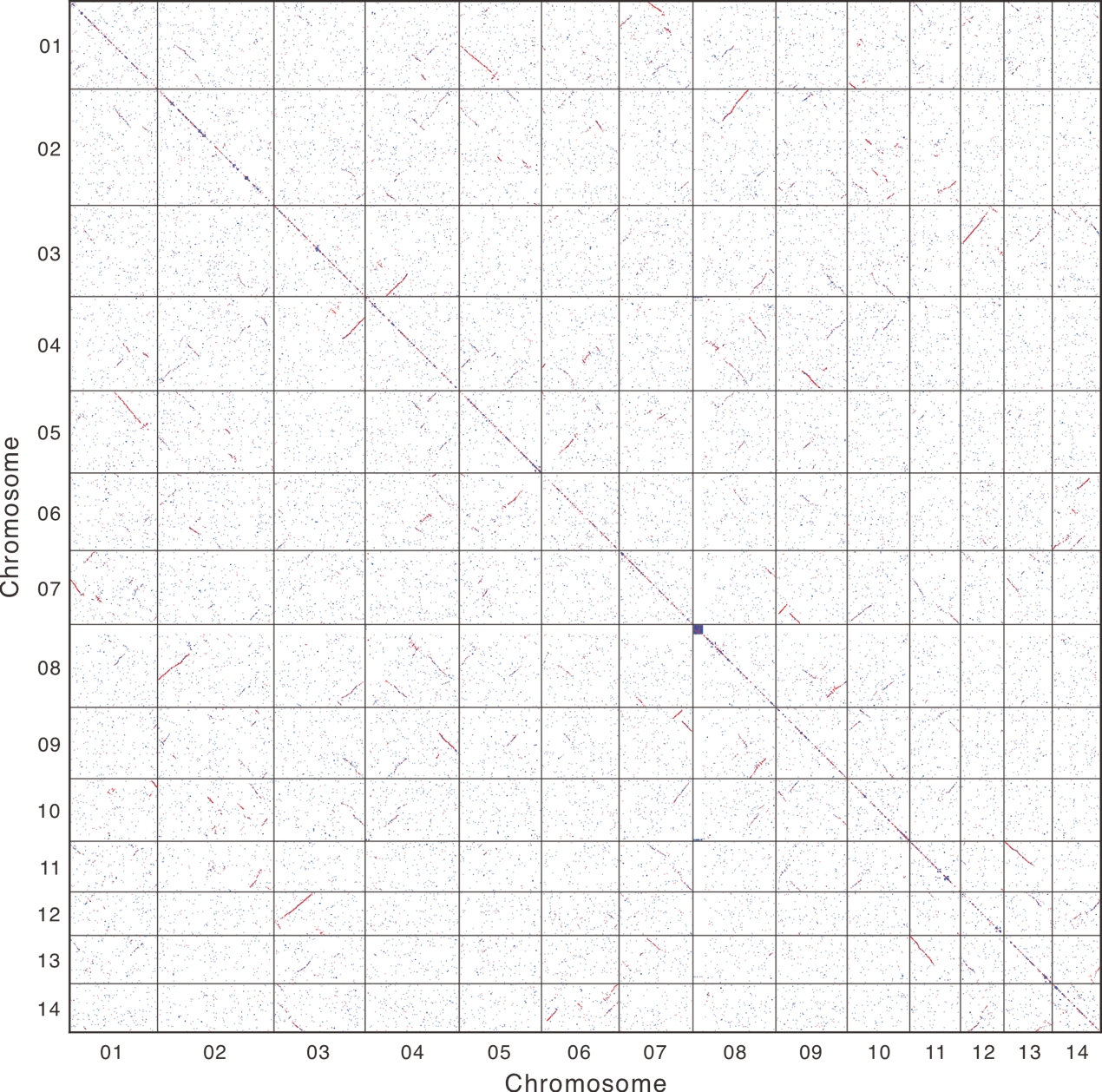


**Figure S3.** Homologous dot plot within the genome (hap1) of monk fruit, exhibiting signals of whole genome duplication (WGD) events.


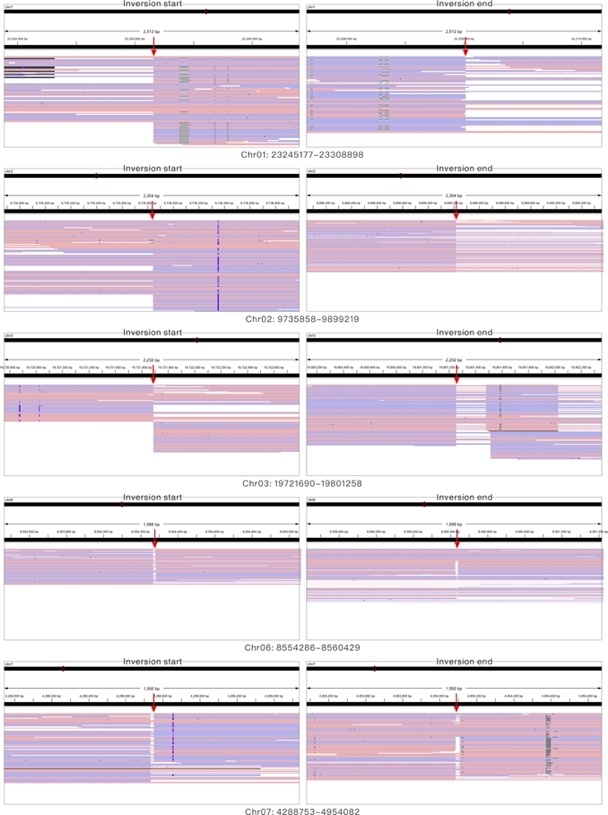

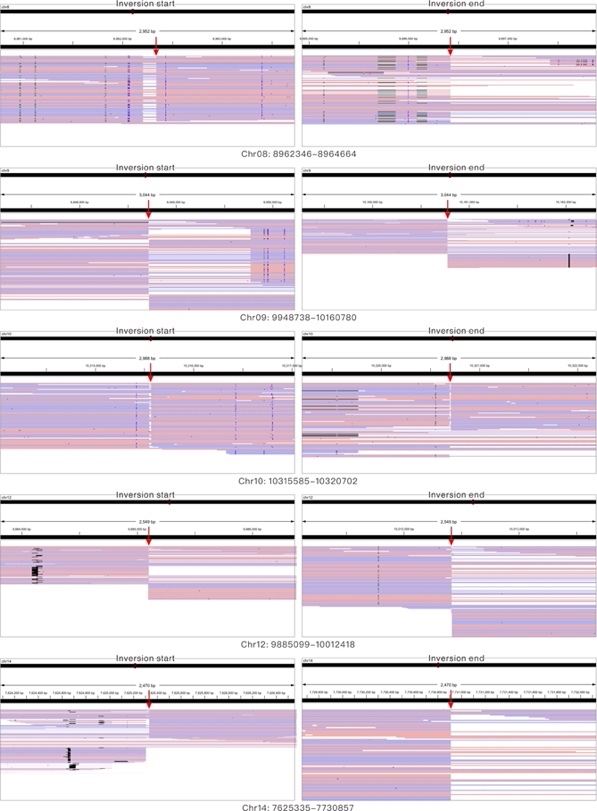


**Figure S4.** Inversions that detected in the haplotype-resolved genome of monk fruit confirmed by inspecting the breakpoints of HiFi reads mapped to the reference genome (Hap1). Red arrows indicate boundaries of the inversions.


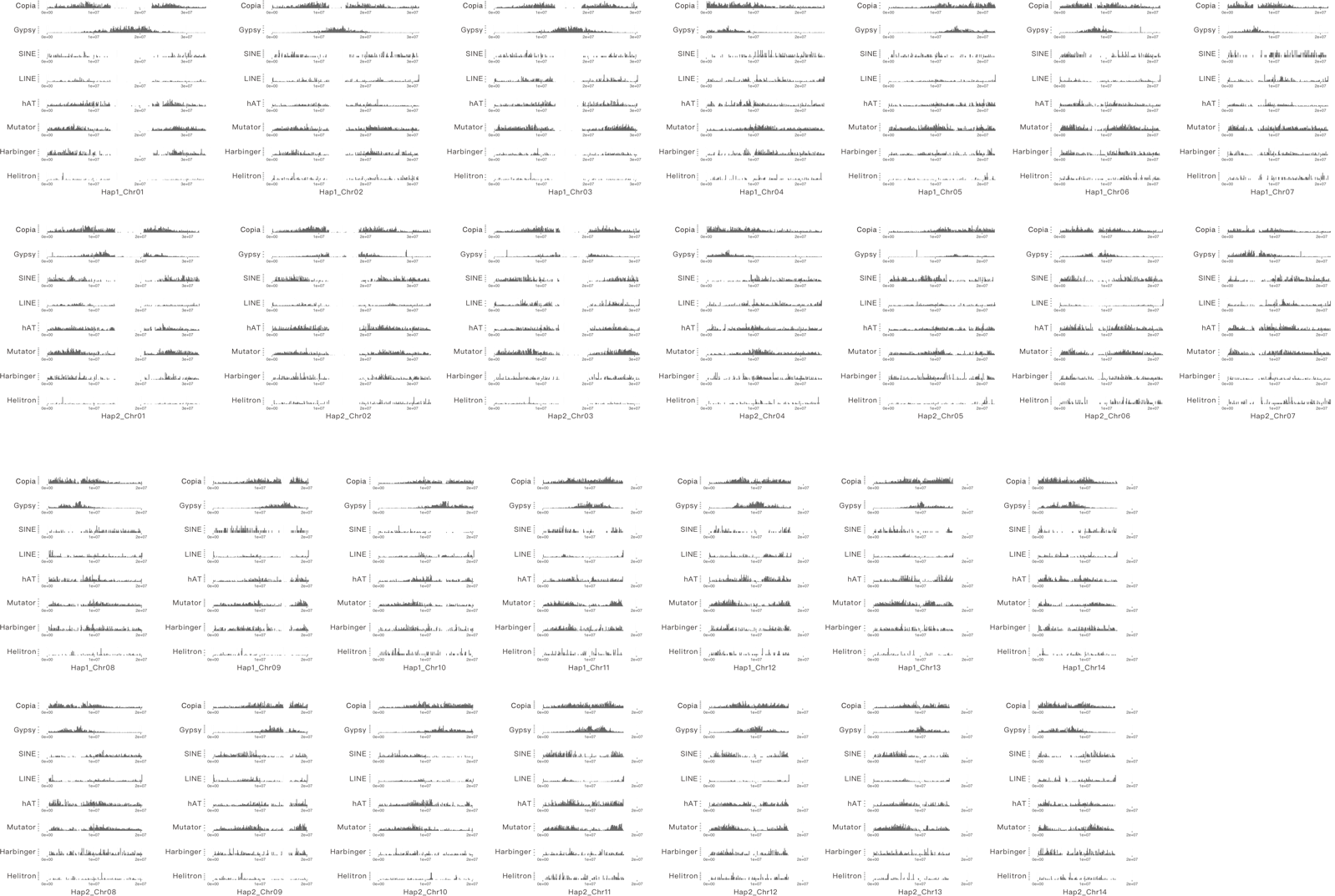


**Figure S5.** Distributions of different types of TEs (Copia, Gypsy, SINE, LINE, hAT, Mutator, Harbinger, and Helitron) on chromosomes of monk fruit genome (Hap1 and Hap2).


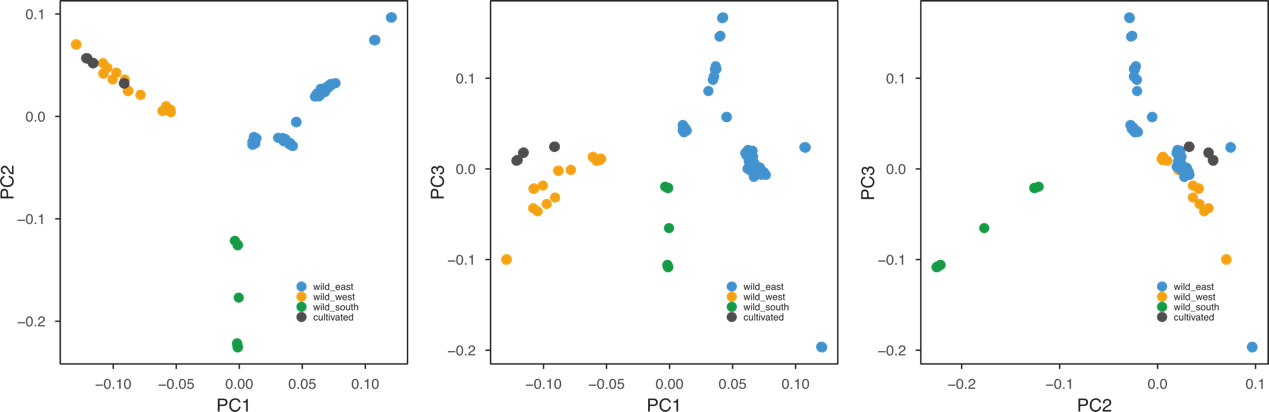


**Figure S6.** Principal component analysis (PCA) of cultivated and wild populations of monk fruit. The three groups within the wild population correspond separately to the clusters revealed in structure analysis in Figure 3.


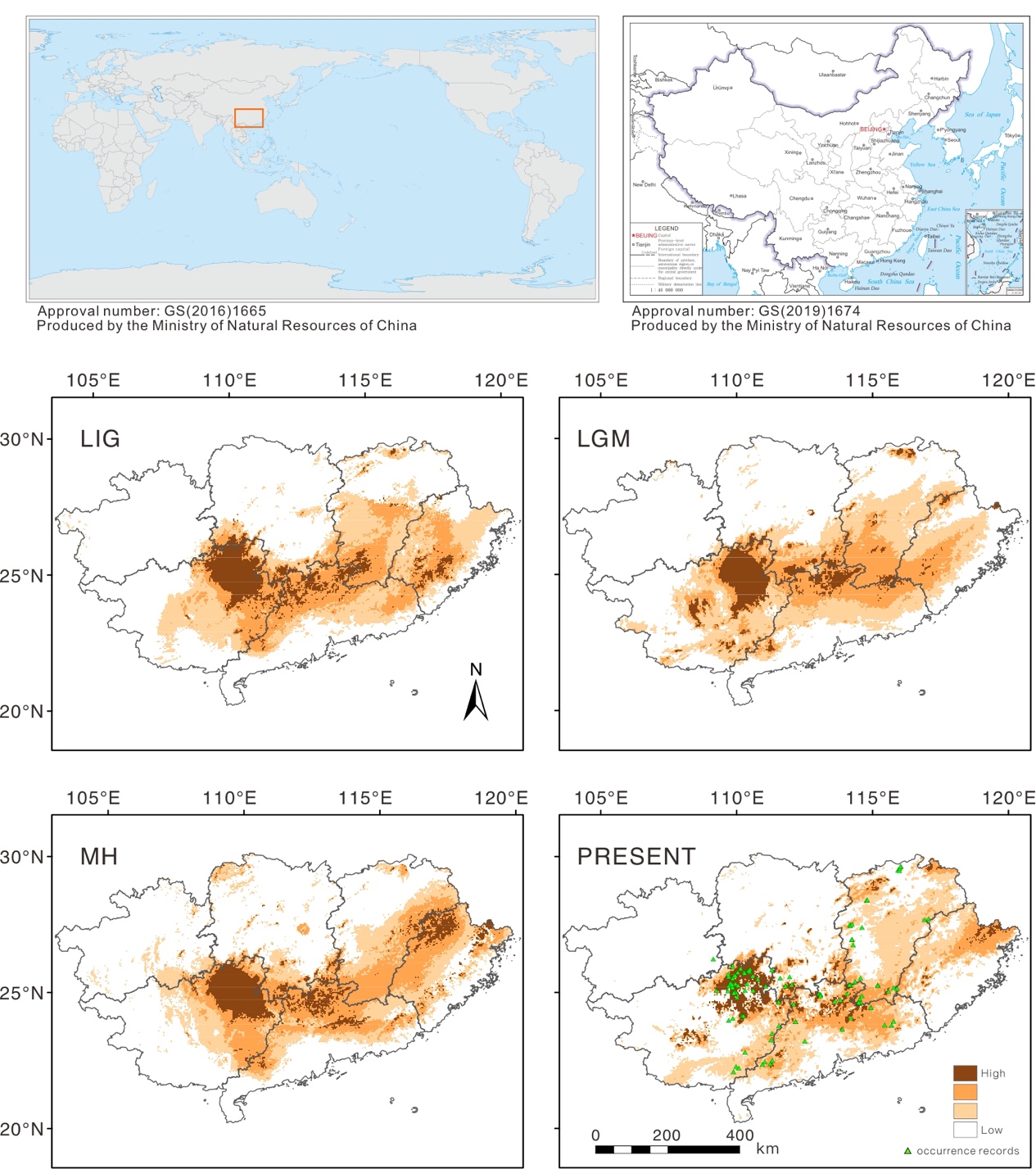


**Figure S7.** Ecological niche modeling of monk fruit across four historical climatic periods (the Present, the MH, the LGM, and the LIG) using MaxEnt. Base map downloaded from the Ministry of Natural Resources of China with an approval number GS(2016)1665 and GS(2019)1674 (<http://bzdt.ch.mnr.gov.cn/index.html>).


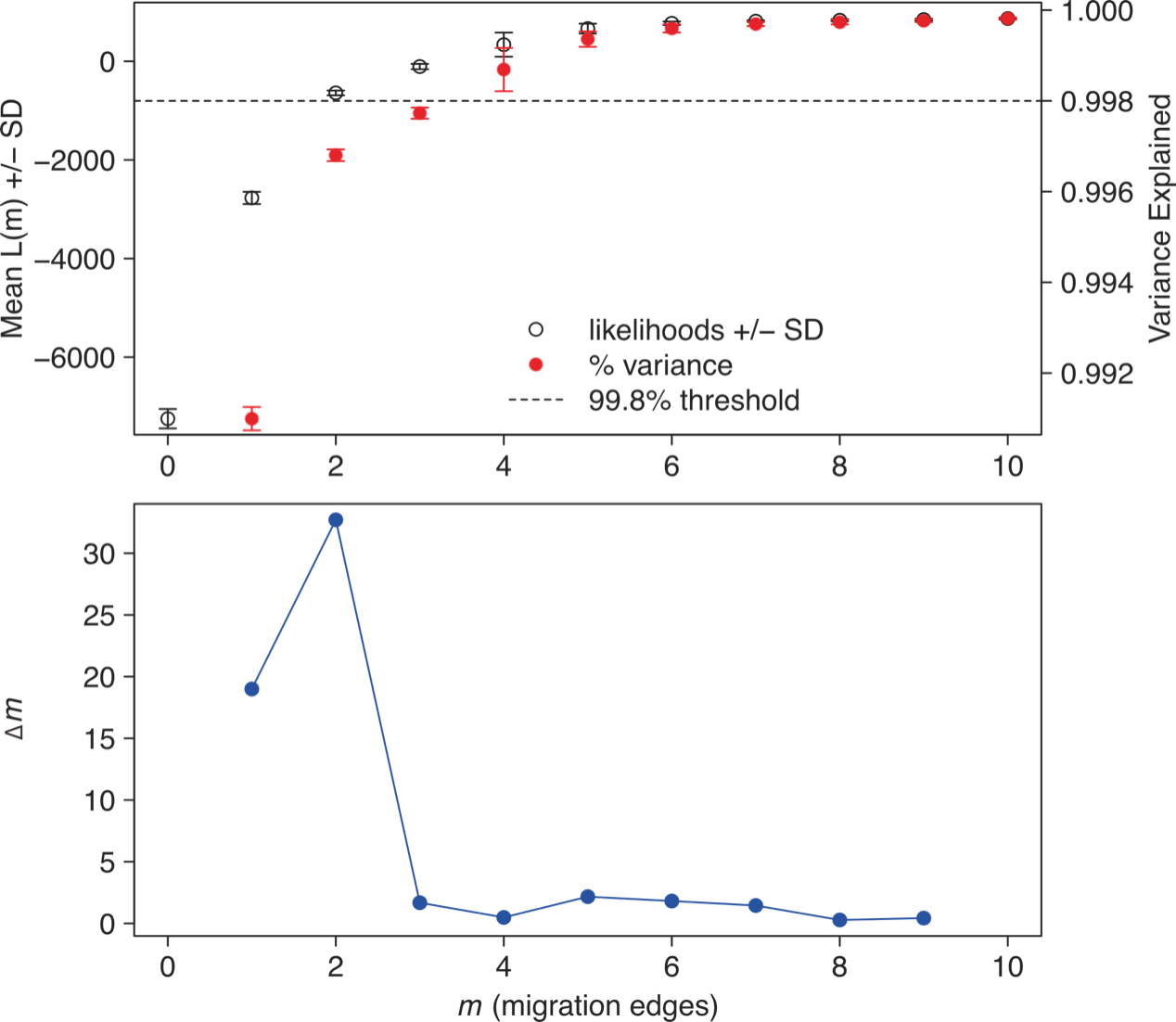


**Figure S8.** Estimation of the optimum number of migration edges (*m*) on population trees using Δm calculated by R package OptM.


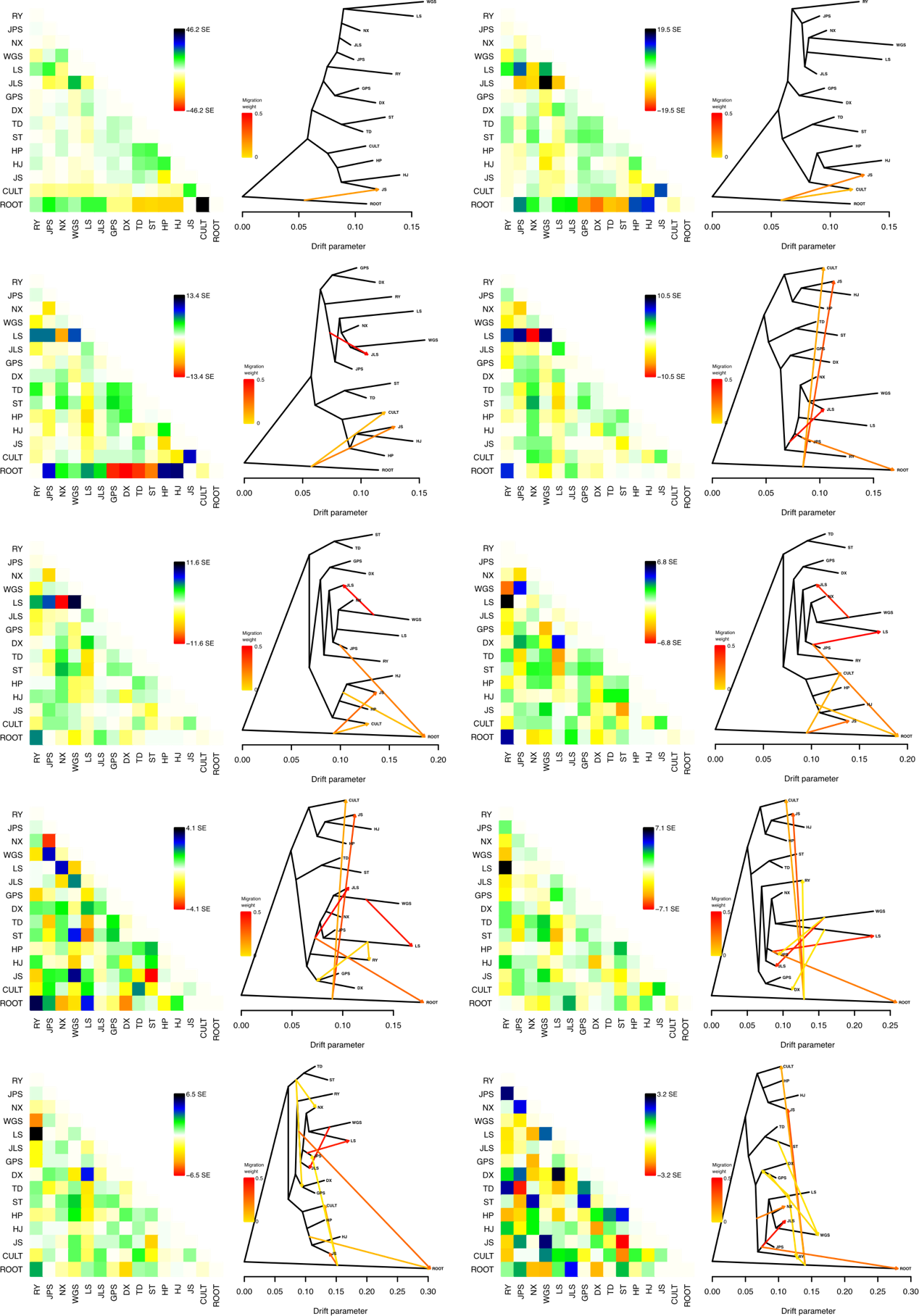


**Figure S9.** Inference of population splits and mixture events of monk fruit using TreeMix, with migration edges (*m*) ranging from 0 to 10 and *S. siamensis* as the root taxon.


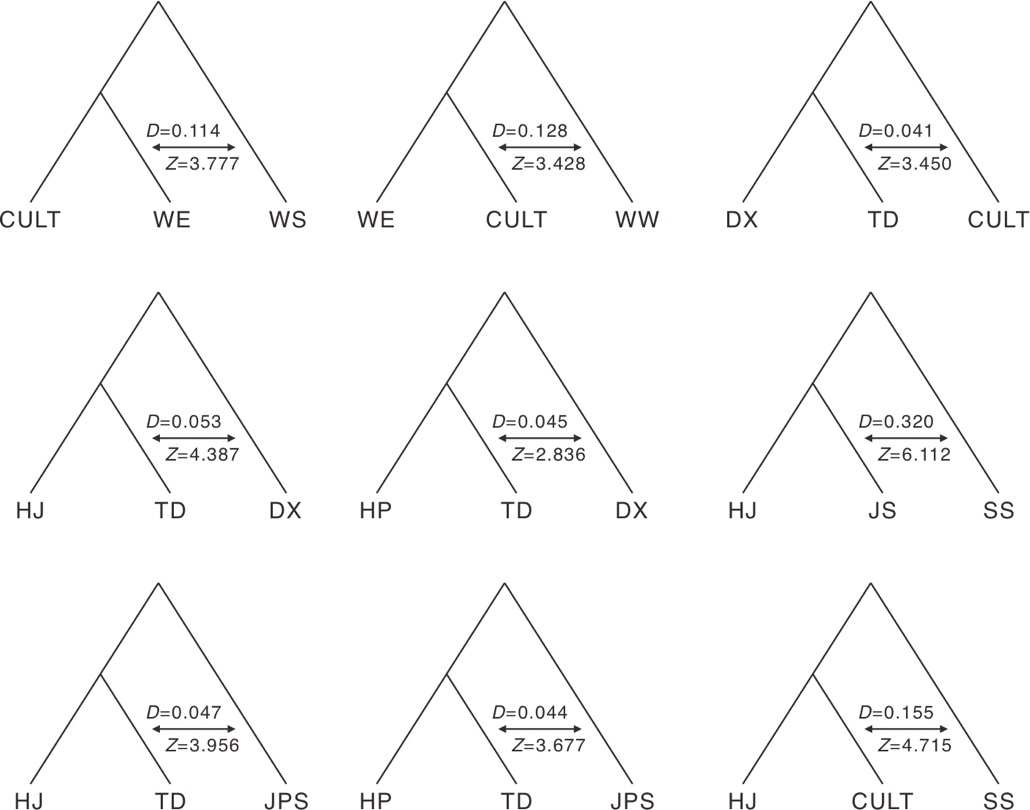


**Figure S10.** Gene flow among populations or groups estimated using ABBA-BABA tests in Dsuite. The arrows indicate gene flow among populations or groups. *D* statistics and *Z* scores are shown above and under the arrows. CULT and SS represent the cultivated population of monk fruit and the related species *Siraitia siamensis*, respectively.


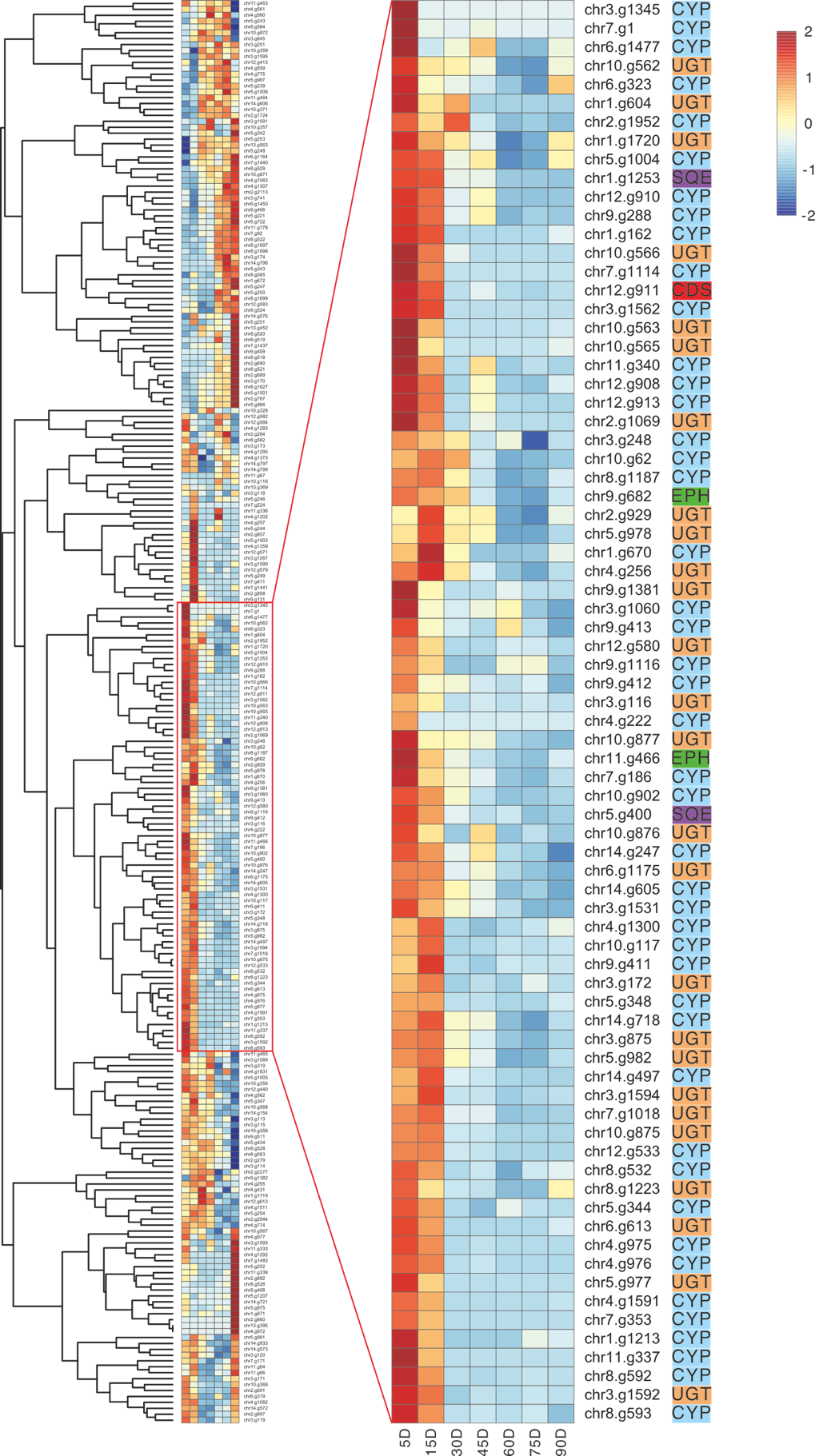


**Figure S11.** Expression patterns of genes related to mogroside biosynthesis across seven fruit development stages of monk fruit. The genes zoomed in the right panel form a significant co-expression module.


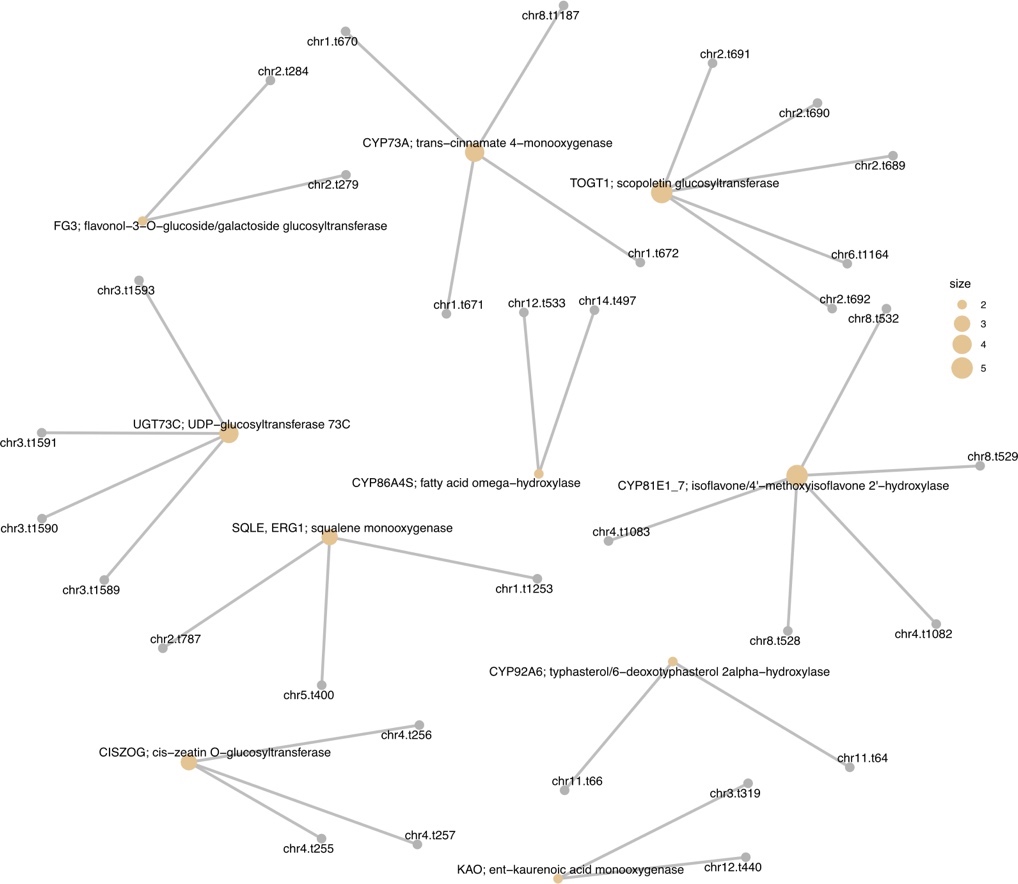


**Figure S12.** Gene-concept network between genes in the mogroside biosynthesis pathway and KEGG pathways constructed using R package enrichplot. The yellow dots indicate KEGG pathways, and the gray dots indicate a gene.


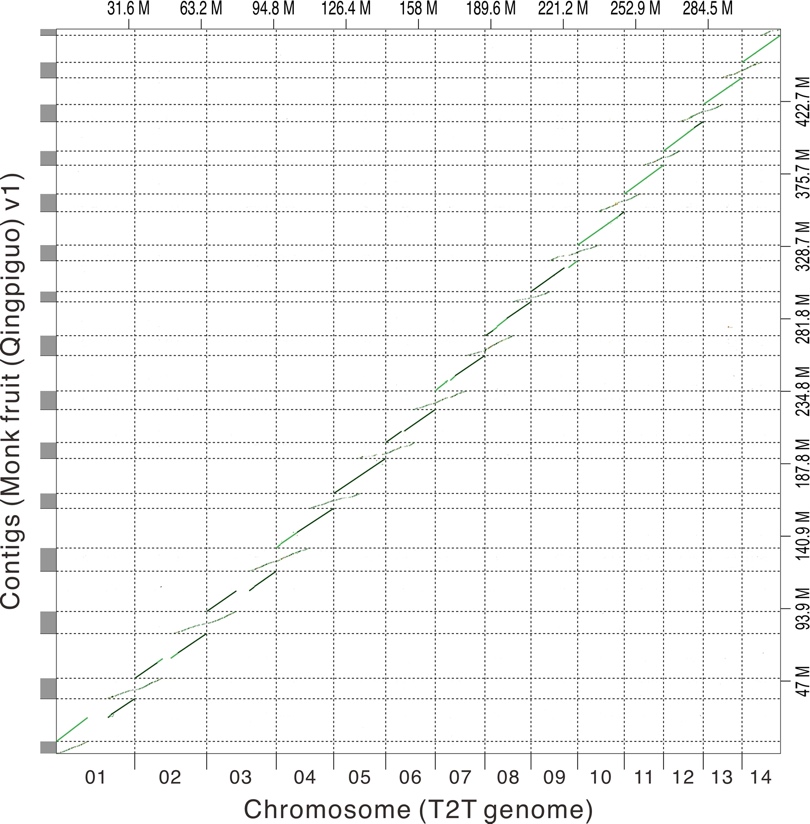


**Figure S13.** The synteny between the T2T genome (Hap1) assembled in the present study and the genome comprising 4182 contigs reported by Xia et al. (2018).


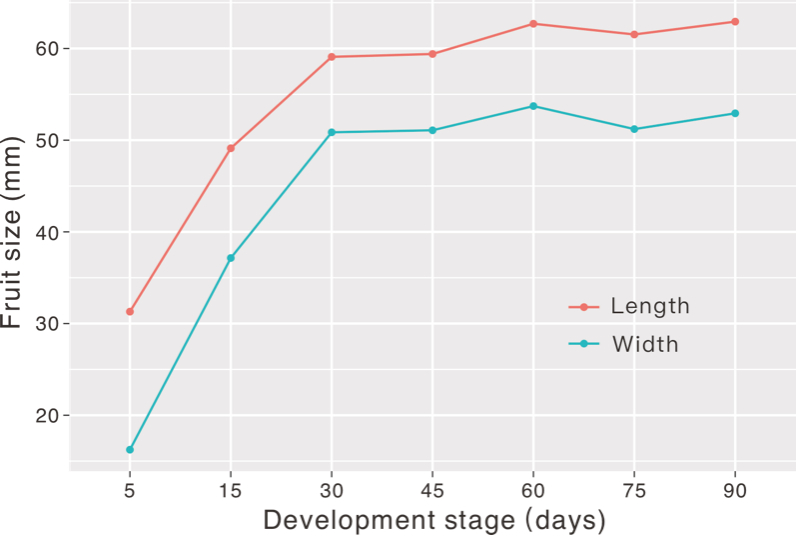


**Figure S14.** Changes in fruit size (length and width) of monk fruit during fruit development.

**Table S1.** Statistics for genome assemblies of the two haplotypes (Hap1 and Hap2) of monk fruit.

|  | Draft genome (HiFi+ONT+Hi-C) | |  | Final T2T genome | |
| --- | --- | --- | --- | --- | --- |
|  | Hap1 | Hap2 |  | Hap 1 | Hap 2 |
| Min sequence length | 1857 | 116 |  | 16247522 | 16817251 |
| Max sequence length | 33844508 | 34154831 |  | 33868581 | 34207398 |
| Total sequence number | 298 | 122 |  | 14 | 14 |
| N20 | 31015167 | 31366007 |  | 32524480 | 31377184 |
| N20 Number | 3 | 2 |  | 2 | 2 |
| N50 | 21876059 | 21650309 |  | 22074204 | 21671274 |
| N50 Number | 7 | 6 |  | 6 | 6 |
| N90 | 10104890 | 9652918 |  | 16438370 | 16912139 |
| N90 Number | 14 | 14 |  | 13 | 13 |
| N number | 0 | 0 |  | 0 | 0 |
| N rate | 0 | 0 |  | 0 | 0 |
| Total sequence length | 335332175 | 326220914 |  | 316205022 | 316070445 |
| GC content % | 34.26 | 34.25 |  | 33.68 | 33.65 |
| Sequences greater than 1kb | 298 | 121 |  | 14 | 14 |

**Table S2.** Statistics for telomeres and centromeres in the two assembled haplotypes (Hap1 and Hap2) of monk fruit.

| Haplotype | Chromosome | Telomeres1 | | |  | Telomeres2 | | |  | Centromeres | | |
| --- | --- | --- | --- | --- | --- | --- | --- | --- | --- | --- | --- | --- |
|  |  | Start | End | Length |  | Start | End | Length |  | Start | End | Length |
| Hap1 | Chr01 | 1 | 25,760 | 25,760 |  | 32,507,370 | 32,524,480 | 17,111 |  | 14,481,624 | 20,961,329 | 6,479,706 |
|  | Chr02 | 1 | 13,300 | 13,300 |  | 33,854,730 | 33,868,581 | 13,852 |  | 12,177,857 | 18,654,782 | 6,476,926 |
|  | Chr03 | 1 | 16,800 | 16,800 |  | 31,037,090 | 31,048,844 | 11,755 |  | 14,350,000 | 20,454,578 | 6,104,579 |
|  | Chr04 | 1 | 12,390 | 12,390 |  | 24,505,530 | 24,515,689 | 10,160 |  | 3,992,077 | 4,867,346 | 875,270 |
|  | Chr05 | 1 | 25,340 | 25,340 |  | 22,704,220 | 22,722,796 | 18,577 |  | 13,979,952 | 14,931,450 | 951,499 |
|  | Chr06 | 1 | 19,950 | 19,950 |  | 22,047,340 | 22,074,204 | 26,865 |  | 6,828,613 | 8,381,982 | 1,553,370 |
|  | Chr07 | 1 | 17,360 | 17,360 |  | 21,890,680 | 21,903,318 | 12,639 |  | 5,579,810 | 6,747,913 | 1,168,104 |
|  | Chr08 | 1 | 11,760 | 11,760 |  | 20,297,270 | 20,304,924 | 7,655 |  | 6,307,393 | 7,722,995 | 1,415,603 |
|  | Chr09 | 1 | 7,910 | 7,910 |  | 20,168,120 | 20,182,081 | 13,962 |  | 14,916,661 | 16,493,947 | 1,577,287 |
|  | Chr10 | 1 | 15,470 | 15,470 |  | 20,212,290 | 20,223,360 | 11,071 |  | 12,980,427 | 13,594,084 | 613,658 |
|  | Chr11 | 1 | 27,790 | 27,790 |  | 17,124,590 | 17,135,767 | 11,178 |  | 9,563,864 | 10,459,070 | 895,207 |
|  | Chr12 | 1 | 19,180 | 19,180 |  | 16,988,020 | 17,015,086 | 27,067 |  | 10,175,047 | 11,255,957 | 1,080,911 |
|  | Chr13 | 1 | 22,330 | 22,330 |  | 16,233,770 | 16,247,522 | 13,753 |  | 10,160,093 | 10,652,571 | 492,479 |
|  | Chr14 | 1 | 10,780 | 10,780 |  | 16,422,840 | 16,438,370 | 15,531 |  | 6,360,146 | 6,651,911 | 291,766 |
|  |  |  |  |  |  |  |  |  |  |  |  |  |
| Hap2 | Chr01 | 1 | 25,760 | 25,760 |  | 34,191,150 | 34,207,398 | 16,249 |  | 13,559,622 | 22,487,467 | 8,927,846 |
|  | Chr02 | 1 | 13,860 | 13,860 |  | 31,363,360 | 31,377,184 | 13,825 |  | 12,225,000 | 15,675,000 | 3,450,001 |
|  | Chr03 | 1 | 16,800 | 16,800 |  | 30,383,010 | 30,394,760 | 11,751 |  | 12,840,620 | 18,632,409 | 5,791,790 |
|  | Chr04 | 1 | 11,970 | 11,970 |  | 25,034,870 | 25,045,018 | 10,149 |  | 4,257,539 | 4,748,856 | 491,318 |
|  | Chr05 | 1 | 25,340 | 25,340 |  | 22,692,180 | 22,710,815 | 18,636 |  | 13,982,525 | 14,934,023 | 951,499 |
|  | Chr06 | 1 | 19,880 | 19,880 |  | 21,619,080 | 21,629,083 | 10,004 |  | 6,935,435 | 8,116,516 | 1,181,082 |
|  | Chr07 | 1 | 13,090 | 13,090 |  | 21,658,560 | 21,671,274 | 12,715 |  | 5,500,000 | 6,550,000 | 1,050,001 |
|  | Chr08 | 1 | 11,410 | 11,410 |  | 20,090,140 | 20,099,932 | 9,793 |  | 6,349,871 | 7,144,039 | 794,169 |
|  | Chr09 | 1 | 7,560 | 7,560 |  | 20,355,510 | 20,369,535 | 14,026 |  | 14,452,850 | 16,659,398 | 2,206,549 |
|  | Chr10 | 1 | 15,400 | 15,400 |  | 20,332,970 | 20,349,271 | 16,302 |  | 13,488,025 | 14,558,897 | 1,070,873 |
|  | Chr11 | 1 | 15,470 | 15,470 |  | 17,114,090 | 17,132,689 | 18,600 |  | 9,306,712 | 9,778,553 | 471,842 |
|  | Chr12 | 1 | 15,050 | 15,050 |  | 17,340,190 | 17,354,096 | 13,907 |  | 9,945,024 | 11,241,501 | 1,296,478 |
|  | Chr13 | 1 | 22,050 | 22,050 |  | 16,895,900 | 16,912,139 | 16,240 |  | 10,000,000 | 10,450,000 | 450,001 |
|  | Chr14 | 1 | 14,630 | 14,630 |  | 16,801,750 | 16,817,251 | 15,502 |  | 6,480,000 | 6,875,000 | 395,001 |

**Table S3.** Statistics for repetitive sequences in the monk fruit genome assembled in the present study.

|  | | Hap1 | | |  | Hap2 | | |
| --- | --- | --- | --- | --- | --- | --- | --- | --- |
| Elements | | Number of elements | Length (bp) | Percentage of Genome (%) |  | Number of elements | Length (bp) | Percentage of Genome (%) |
| **Retroelements** |  | 141,317 | 76,074,593 | 24.06 |  | 145,272 | 76,489,222 | 24.2 |
|  | **SINEs** | 6,241 | 494,300 | 0.16 |  | 2,009 | 184,796 | 0.06 |
|  | **LINEs** | 25,779 | 8,245,892 | 2.61 |  | 23,114 | 7,868,710 | 2.49 |
|  | **LTR elements** | 109,297 | 67,334,401 | 21.29 |  | 120,149 | 68,435,716 | 21.65 |
|  | Ty1/Copia | 41,666 | 26,736,604 | 8.46 |  | 40,342 | 27,997,762 | 8.86 |
|  | Gypsy/DIRS1 | 59,283 | 37,830,745 | 11.96 |  | 69,894 | 37,040,422 | 11.72 |
| **DNA transposons** |  | 84,053 | 19,467,094 | 6.16 |  | 69,823 | 17,451,351 | 5.52 |
|  | **hobo-Activator** | 18,316 | 4,899,351 | 1.55 |  | 13,871 | 4,060,307 | 1.28 |
|  | **MULE-MuDR** | 46,735 | 8,877,030 | 2.81 |  | 39,828 | 7,928,251 | 2.51 |
|  | **Tourist/Harbinger** | 5,297 | 1,176,758 | 0.37 |  | 7,484 | 1,670,140 | 0.53 |
| **Rolling-circles** |  | 2,388 | 474,207 | 0.15 |  | 2,041 | 396,344 | 0.13 |
| **Unclassified** |  | 323,868 | 71,529,686 | 22.62 |  | 463,246 | 72,939,921 | 23.08 |
| **other** |  | 3,360 | 456,837 | 0.15 |  | 2,071 | 486,253 | 0.16 |

**Table S4.** KEGG pathways enriched in the expansion gene families during the evolution of monk fruit genome.

| ID | Description | Rich Factor | *P* value | Count |
| --- | --- | --- | --- | --- |
| map00940 | Phenylpropanoid biosynthesis | 0.45394737 | 7.92102E-36 | 69 |
| map00909 | Sesquiterpenoid and triterpenoid biosynthesis | 0.63636364 | 1.36777E-10 | 14 |
| map00360 | Phenylalanine metabolism | 0.43902439 | 1.14905E-09 | 18 |
| map04075 | Plant hormone signal transduction | 0.17557252 | 8.19351E-07 | 46 |
| map00480 | Glutathione metabolism | 0.25316456 | 5.57179E-06 | 20 |
| map00941 | Flavonoid biosynthesis | 0.43478261 | 7.27086E-06 | 10 |
| map00908 | Zeatin biosynthesis | 0.37931034 | 1.22274E-05 | 11 |
| map00945 | Stilbenoid, diarylheptanoid and gingerol biosynthesis | 0.46666667 | 1.06912E-04 | 7 |
| map00196 | Photosynthesis - antenna proteins | 0.4 | 1.27616E-04 | 8 |
| map01040 | Biosynthesis of unsaturated fatty acids | 0.36363636 | 2.78276E-04 | 8 |
| map00330 | Arginine and proline metabolism | 0.23529412 | 8.89579E-04 | 12 |
| map00040 | Pentose and glucuronate interconversions | 0.16666667 | 1.03366E-02 | 14 |
| map00130 | Ubiquinone and other terpenoid-quinone biosynthesis | 0.15909091 | 7.53248E-02 | 7 |
| map00750 | Vitamin B6 metabolism | 0.21428571 | 1.10053E-01 | 3 |
| map00290 | Valine, leucine and isoleucine biosynthesis | 0.2 | 1.29308E-01 | 3 |
| map04626 | Plant-pathogen interaction | 0.10852713 | 2.04256E-01 | 14 |
| map00460 | Cyanoamino acid metabolism | 0.12903226 | 2.67780E-01 | 4 |
| map00592 | alpha-Linolenic acid metabolism | 0.11627907 | 3.00900E-01 | 5 |
| map00450 | Selenocompound metabolism | 0.125 | 4.00282E-01 | 2 |
| map00380 | Tryptophan metabolism | 0.09756098 | 4.65670E-01 | 4 |

**Table S5.** KEGG pathways enriched in genes under the specific chromosomal segments in Hap1 and Hap2 of monk fruit genome.

|  | ID | Description | Rich Factor | *P* value | Count |
| --- | --- | --- | --- | --- | --- |
| Hap1 | map00040 | Pentose and glucuronate interconversions | 0.05952 | 0.00041 | 5 |
|  | map00520 | Amino sugar and nucleotide sugar metabolism | 0.03226 | 0.01465 | 4 |
|  | map00190 | Oxidative phosphorylation | 0.02857 | 0.04682 | 3 |
|  | map04122 | Sulfur relay system | 0.10000 | 0.07519 | 1 |
|  | map04141 | Protein processing in endoplasmic reticulum | 0.01911 | 0.12089 | 3 |
|  | map00270 | Cysteine and methionine metabolism | 0.02299 | 0.14600 | 2 |
|  | map00790 | Folate biosynthesis | 0.04000 | 0.17781 | 1 |
|  | map00920 | Sulfur metabolism | 0.03704 | 0.19063 | 1 |
|  | map00020 | Citrate cycle (TCA cycle) | 0.02326 | 0.28646 | 1 |
|  | map00052 | Galactose metabolism | 0.02326 | 0.28646 | 1 |
|  | map00970 | Aminoacyl-tRNA biosynthesis | 0.02174 | 0.30316 | 1 |
|  | map03010 | Ribosome | 0.01154 | 0.32935 | 3 |
|  | map00630 | Glyoxylate and dicarboxylate metabolism | 0.01754 | 0.36122 | 1 |
|  | map04146 | Peroxisome | 0.01449 | 0.41923 | 1 |
|  | map03015 | mRNA surveillance pathway | 0.01124 | 0.50475 | 1 |
|  | map04120 | Ubiquitin mediated proteolysis | 0.01064 | 0.52415 | 1 |
|  |  |  |  |  |  |
| Hap2 | map00040 | Pentose and glucuronate interconversions | 0.05435 | 0.00037 | 5 |
|  | map00592 | alpha-Linolenic acid metabolism | 0.04762 | 0.03403 | 2 |
|  | map04122 | Sulfur relay system | 0.10000 | 0.06768 | 1 |
|  | map04141 | Protein processing in endoplasmic reticulum | 0.01935 | 0.09152 | 3 |
|  | map00270 | Cysteine and methionine metabolism | 0.02273 | 0.12455 | 2 |
|  | map00790 | Folate biosynthesis | 0.04000 | 0.16099 | 1 |
|  | map00920 | Sulfur metabolism | 0.03704 | 0.17272 | 1 |
|  | map00520 | Amino sugar and nucleotide sugar metabolism | 0.01575 | 0.22146 | 2 |
|  | map00052 | Galactose metabolism | 0.02326 | 0.26110 | 1 |
|  | map03010 | Ribosome | 0.01158 | 0.26905 | 3 |
|  | map00970 | Aminoacyl-tRNA biosynthesis | 0.02128 | 0.28173 | 1 |
|  | map00630 | Glyoxylate and dicarboxylate metabolism | 0.01786 | 0.32613 | 1 |
|  | map04146 | Peroxisome | 0.01429 | 0.38999 | 1 |
|  | map03015 | mRNA surveillance pathway | 0.01124 | 0.46739 | 1 |
|  | map04120 | Ubiquitin mediated proteolysis | 0.01064 | 0.48612 | 1 |
|  | map04075 | Plant hormone signal transduction | 0.00766 | 0.55222 | 2 |

**Table S6.** Summary of whole-genome resequencing data for monk fruit samples on the Illumina platform.

| Sample | Mean depth (×) | Coverage (%) | Mapping rate (%) |  | Sample | Mean depth (×) | Coverage (%) | Mapping rate (%) |
| --- | --- | --- | --- | --- | --- | --- | --- | --- |
| DX01 | 24.98 | 90.12 | 89.64 |  | LS01 | 23.78 | 89.37 | 91.11 |
| DX02 | 25.49 | 90.27 | 91.81 |  | LS02 | 25.07 | 89.39 | 91.1 |
| DX03 | 25.09 | 90.29 | 90.1 |  | LS03 | 25.47 | 89.56 | 93.68 |
| DX04 | 24.13 | 90.09 | 90.06 |  | LS04 | 24.74 | 89.44 | 94.37 |
| DX05 | 29.03 | 90.14 | 92.84 |  | LS05 | 25.32 | 89.47 | 93.3 |
| DX06 | 26.15 | 90.41 | 91.76 |  | LS06 | 27.07 | 89.61 | 91.87 |
| DX07 | 27.07 | 90.48 | 90.53 |  | LS07 | 25.51 | 89.62 | 91.37 |
| DX08 | 24.95 | 89.8 | 91.82 |  | LS08 | 27.32 | 89.7 | 94.66 |
| DX09 | 24.68 | 89.98 | 90.51 |  | LS09 | 27.25 | 89.51 | 92.4 |
| DX10 | 39.09 | 90.48 | 90.76 |  | LS10 | 25.69 | 89.66 | 93.39 |
| GPS01 | 23.16 | 90.5 | 87.33 |  | NX01 | 24.08 | 89.62 | 89.54 |
| GPS02 | 26.06 | 90.76 | 91.7 |  | NX02 | 26.59 | 89.72 | 90.41 |
| GPS03 | 25.01 | 90.63 | 91.47 |  | NX03 | 26.62 | 89.56 | 91.53 |
| GPS04 | 25.22 | 90.6 | 89.79 |  | NX04 | 28.98 | 90.52 | 90.1 |
| GPS05 | 24.33 | 90.61 | 88.39 |  | NX05 | 25.22 | 89.7 | 90.72 |
| GPS06 | 26.01 | 90.7 | 89.18 |  | NX06 | 25.4 | 89.83 | 90.13 |
| GPS07 | 31.64 | 90.99 | 89.15 |  | NX07 | 24.89 | 89.91 | 91.14 |
| GPS08 | 24.98 | 90.52 | 90.13 |  | NX08 | 28.62 | 89.82 | 90.67 |
| GPS09 | 25.09 | 90.49 | 91.04 |  | NX09 | 25.78 | 89.77 | 92.82 |
| GPS10 | 26.14 | 90.52 | 88.5 |  | NX10 | 27.62 | 90.25 | 89.61 |
| HJ01 | 33.04 | 91.47 | 86.76 |  | RY01 | 25.78 | 90.06 | 95.3 |
| HJ02 | 25.03 | 91.14 | 88.36 |  | RY02 | 26.25 | 90.13 | 94.53 |
| HJ03 | 25.35 | 91.01 | 87.03 |  | RY03 | 25.56 | 89.95 | 92.33 |
| HJ04 | 26.55 | 91.28 | 90.62 |  | RY04 | 25.83 | 89.9 | 95.42 |
| HJ05 | 25.46 | 91.22 | 90.19 |  | RY05 | 23.98 | 89.87 | 95 |
| HJ06 | 26.81 | 91.21 | 90.74 |  | RY06 | 26.9 | 90.06 | 93.64 |
| HJ07 | 24.52 | 91.05 | 89.12 |  | ST01 | 52.13 | 92.26 | 92.85 |
| HJ08 | 25.16 | 91.03 | 89.12 |  | ST02 | 39.2 | 92 | 92.86 |
| HJ09 | 24.72 | 91.03 | 87.63 |  | ST03 | 62.99 | 92.35 | 92.58 |
| HJ10 | 25.56 | 91.2 | 89.93 |  | ST04 | 37.91 | 91.74 | 93.88 |
| HP01 | 25.15 | 92.36 | 89.19 |  | ST05 | 52.61 | 92.24 | 92.84 |
| HP02 | 24.46 | 92.38 | 89.26 |  | ST06 | 46.8 | 92.06 | 93 |
| HP04 | 24.65 | 91.63 | 87.84 |  | ST07 | 44.9 | 92.08 | 92.8 |
| HP05 | 25.58 | 91.76 | 88.86 |  | ST08 | 58.7 | 92.37 | 94.03 |
| HP06 | 26.24 | 91.9 | 88.26 |  | ST09 | 35.67 | 91.81 | 92.83 |
| HP07 | 25 | 91.75 | 92.2 |  | ST10 | 54.33 | 92.72 | 93.04 |
| HP08 | 24.85 | 91.21 | 93.68 |  | TD01 | 24.36 | 91.63 | 90.05 |
| HP09 | 26.06 | 91.84 | 90.7 |  | TD02 | 25.22 | 90.9 | 90.43 |
| HP10 | 27.76 | 91.91 | 88.53 |  | TD03 | 26.51 | 91.81 | 92.7 |
| JLS01 | 24.63 | 90.18 | 93.16 |  | TD04 | 26.28 | 91.86 | 92.69 |
| JLS02 | 23.42 | 89.74 | 94.23 |  | TD05 | 26.97 | 91.89 | 92.71 |
| JLS03 | 24.89 | 90.1 | 94.35 |  | TD06 | 26.82 | 91.92 | 93.19 |
| JLS04 | 23.91 | 89.78 | 92.28 |  | TD07 | 26.62 | 91.42 | 92.93 |
| JLS05 | 27.11 | 90.22 | 92.55 |  | TD08 | 25.71 | 91.34 | 91.56 |
| JLS06 | 25.99 | 89.83 | 91.15 |  | TD09 | 25.1 | 91.92 | 92.77 |
| JLS07 | 26.83 | 90.09 | 92.48 |  | TD10 | 25.56 | 91.72 | 92.22 |
| JLS08 | 24.6 | 89.73 | 94.77 |  | WGS01 | 29.65 | 89.63 | 92.25 |
| JLS09 | 23.42 | 90.17 | 93.45 |  | WGS02 | 24.58 | 89.29 | 94.37 |
| JLS10 | 26.37 | 89.72 | 94.16 |  | WGS03 | 27.19 | 89.52 | 91.48 |
| JPS01 | 26.63 | 90.03 | 92.04 |  | WGS04 | 28.8 | 89.52 | 92.45 |
| JPS02 | 25.34 | 89.81 | 92.6 |  | WGS05 | 26.16 | 89.24 | 92.08 |
| JPS03 | 27.03 | 89.99 | 93.02 |  | WGS06 | 27.21 | 89.34 | 91.25 |
| JPS04 | 27.8 | 90.18 | 92.61 |  | WGS07 | 24.88 | 89.28 | 92.93 |
| JPS05 | 26.44 | 89.71 | 91.36 |  | WGS08 | 26.57 | 89.45 | 94.94 |
| JPS06 | 24.43 | 89.82 | 90.15 |  | WGS09 | 28.67 | 89.46 | 92.1 |
| JPS07 | 25.45 | 89.55 | 91.37 |  | WGS10 | 26.95 | 89.31 | 91.95 |
| JPS08 | 24.74 | 89.8 | 91.57 |  | YF01 | 26.89 | 93.8 | 90.07 |
| JPS09 | 27.19 | 89.9 | 91.9 |  | YF02 | 24.91 | 92.32 | 89.55 |
| JPS10 | 25.39 | 88.77 | 93.78 |  | YF03 | 25.12 | 92.14 | 88.8 |
| JS01 | 23.37 | 92.39 | 92.26 |  | YF04 | 24.69 | 92.59 | 92.26 |
| JS02 | 25.96 | 92.36 | 90.99 |  | YF05 | 25.98 | 93.43 | 90.28 |
| JS03 | 25.06 | 92.43 | 91.51 |  | YF07 | 24.81 | 93.74 | 90.92 |
| JS04 | 28.95 | 92.67 | 91.02 |  | YF08 | 24.39 | 91.88 | 91.64 |
| JS05 | 28.81 | 92.54 | 91.27 |  | YF09 | 25.51 | 92.52 | 91.26 |
| JS06 | 26.75 | 92.45 | 90.35 |  | YF10 | 25.8 | 93.26 | 89.38 |
| JS07 | 26.65 | 92.58 | 90.88 |  | ZP01 | 48.95 | 94.67 | 90.4 |
| JS08 | 25.79 | 91.42 | 90.5 |  | ZP02 | 46.96 | 97.42 | 91.89 |
| JS09 | 25.03 | 93.73 | 90.84 |  | ZP03 | 42.44 | 93.71 | 93.72 |
| JS10 | 25.84 | 92.46 | 90.97 |  | ZP-HMG1 | 26.51 | 93.32 | 89.38 |
| JX01 | 25.88 | 91.51 | 90.08 |  | ZP-HMG2 | 26.76 | 93.38 | 88.63 |
| JX02 | 25.43 | 91.56 | 92.46 |  | ZP-HMG3 | 36.43 | 93.55 | 89.07 |
| JX03 | 25.53 | 91.57 | 89.84 |  | ZP-QPG1 | 27.57 | 97.49 | 90.5 |
| JX04 | 25.44 | 91.62 | 90.39 |  | ZP-QPG2 | 29.73 | 97.19 | 92.37 |
| JX05 | 25.29 | 91.58 | 92.32 |  | ZP-QPG3 | 26.66 | 96.9 | 87.45 |
| JX06 | 26.11 | 91.9 | 91.43 |  | ZP-QPG4 | 29.22 | 97 | 91.64 |
| JX07 | 24.63 | 91.86 | 92.29 |  | ZP-QPG5 | 25.81 | 96.92 | 90.09 |
| JX08 | 25.74 | 91.33 | 88.99 |  | ZP-QPG6 | 27.19 | 96.9 | 90.6 |
| JX09 | 30.74 | 91.69 | 89.04 |  | ZP-QPG7 | 25.95 | 97.46 | 90.23 |
| JX10 | 25.04 | 91.28 | 92.97 |  | ZP-XZ1 | 34.02 | 94.56 | 89.03 |
| JX11 | 28.94 | 92.02 | 89.76 |  | ZP-XZ2 | 27.21 | 94.04 | 90.83 |
| JX12 | 26.52 | 91.88 | 91.15 |  | ZP-XZ3 | 26.93 | 93.88 | 87.83 |
| JX13 | 24.18 | 91.75 | 88.7 |  | ZP-XZ4 | 26.69 | 93.82 | 89.72 |
| JX14 | 25.43 | 91.54 | 90.61 |  | ZP-XZ5 | 27.28 | 93.96 | 88.44 |
| JX15 | 24.95 | 91.92 | 89.39 |  |  |  |  |  |

**Table S7.** Population genomic statistics for cultivated population (CULT) and wild populations (WE, WS, and WW) of monk fruit.

|  |  | CULT | WE | WS | WW |
| --- | --- | --- | --- | --- | --- |
| *π* | – | 5.02±3.07×10^-3^ | 6.87±3.48×10^-3^ | 5.08±2.82×10^-3^ | 6.18±3.23×10^-3^ |
|  |  |  |  |  |  |
| Tajima’s *D* | – | 2.21±1.09 | 1.65±0.79 | 2.48±0.77 | 1.57±1.01 |
|  |  |  |  |  |  |
| *F*_ST_ | WE | 0.27±0.10 | – | – | – |
|  | WS | 0.37±0.12 | 0.23±0.10 | – | – |
|  | WW | 0.23±0.12 | 0.23±0.09 | 0.30±0.10 | – |

**Table S8.** Candidate genes identified in the potential selective sweeps of cultivated monk fruit.

| Trait | Chromosome | Gene | Start | End | Gene ID | Annotation |
| --- | --- | --- | --- | --- | --- | --- |
| Disease resistance | chr01 | chr1.t399 | 3414659 | 3424160 | RPP5 | defense response to fungus; TIR-NB-LRR receptor-like protein that confers resistance to the pathogen Hyaloperonospora arabidopsis isolate Noco2 (downy mildew disease) |
|  | chr05 | chr5.t1607 | 19011141 | 19016245 | UPF3 | defense response to bacterium |
|  | chr09 | chr9.t1169 | 8609275 | 8658307 | MOM1 | defense response to bacterium |
| Stress resistance | chr01 | chr1.t1274 | 29949314 | 29953122 | NDL2 | response to cold |
|  | chr01 | chr1.t1276 | 29967234 | 29969348 | MYBS3 | Plants over-expressing MYBS3 show increased tolerance to cold stress. |
|  | chr01 | chr1.t1771 | 33744248 | 33745925 | AIRP2 | Plants over-expressing AIRP2 exhibit tolerance to severe drought stress; response to salt stress |
|  | chr04 | chr4.t563 | 14014595 | 14015026 | HS181 | heat acclimation |
|  | chr05 | chr5.t300 | 1940334 | 1942199 | COR27 | response to cold; regulation of photoperiodism, flowering |
|  | chr05 | chr5.t361 | 2310592 | 2311374 | ZAT10 | response to cold; response to salt stress; response to water deprivation; Plants overexpressing ZAT10 show growth retardation and enhanced tolerance to drought, salt, heat and osmotic stresses. Plants silencing ZAT10 show enhanced tolerance to salt and osmotic stresses. |
|  | chr05 | chr5.t1567 | 17232978 | 17233556 | HSP41 | response to heat; response to salt stress |
|  | chr05 | chr5.t1632 | 19574743 | 19581059 | CPL4 | response to salt stress |
|  | chr07 | chr7.t281 | 10494432 | 10495511 | CHIC | response to salt stress; polysaccharide catabolic process |
|  | chr07 | chr7.t393 | 12489817 | 12511447 | HDA15 | response to heat; positive regulation of photomorphogenesis |
|  | chr10 | chr10.t732 | 5278870 | 5280727 | SRM1 | negative regulation of response to salt stress; regulation of leaf morphogenesis; response to water deprivation |
| Carbohydrate biosynthesis | chr01 | chr1.t854 | 13495787 | 13497136 | XXT3 | xyloglucan 6-xylosyltransferase activity |
|  | chr04 | chr4.t552 | 13952395 | 13954906 | F16P1 | fructose metabolic process; sucrose biosynthetic process |
|  | chr05 | chr5.t1603 | 18869827 | 18906154 | OFUT9 | fucose metabolic process; glycosyltransferase activity |
|  | chr05 | chr5.t1695 | 21903204 | 21904438 | OFUT9 | fucose metabolic process; glycosyltransferase activity |
|  | chr06 | chr6.t778 | 14592808 | 14597163 | GALE2 | galactose metabolic process; UDP-glucose 4-epimerase activity |
|  | chr10 | chr10.t520 | 3466496 | 3468832 | SPP2 | sucrose biosynthetic process; Catalyzes the final step of sucrose synthesis |
|  | chr10 | chr10.t718 | 5182931 | 5185837 | AGAL1 | carbohydrate metabolic process |
| Morphogenesis | chr01 | chr1.t867 | 22735727 | 22754651 | RRG | regulation of root meristem growth |
|  | chr02 | chr2.t785 | 6043716 | 6048611 | CGR2 | leaf morphogenesis; positive regulation of developmental growth |
|  | chr03 | chr3.t9 | 106000 | 108386 | NAC50 | flower development; photoperiodism, flowering |
|  | chr04 | chr4.t542 | 13859864 | 13861870 | OPS | regulation of root development; root system development |
|  | chr04 | chr4.t565 | 14019700 | 14026907 | PAS1 | lateral root development; root development |
|  | chr05 | chr5.t1612 | 19162312 | 19172225 | AGL12 | flower development; root development |
|  | chr05 | chr5.t1620 | 19355940 | 19357124 | KIN17 | regulation of developmental growth |
|  | chr09 | chr9.t1168 | 8588958 | 8593332 | WAV3 | root development |
|  | chr10 | chr10.t717 | 5178053 | 5182174 | AGAL2 | leaf morphogenesis; positive regulation of flower development; response to fungus |
|  | chr10 | chr10.t724 | 5218463 | 5223413 | FYPP | negative regulation of flower development |
| DNA repair | chr10 | chr10.t640 | 4453707 | 4460751 | MUS81 | DNA damage response; DNA repair |

**Table S9.** KEGG pathways enriched in genes under potential selective sweeps in cultivated monk fruit.

| ID | Description | Rich Factor | *P* value | Count |
| --- | --- | --- | --- | --- |
| map00906 | Carotenoid biosynthesis | 0.09677 | 0.00485 | 3 |
| map00230 | Purine metabolism | 0.05000 | 0.01201 | 4 |
| map01040 | Biosynthesis of unsaturated fatty acids | 0.09091 | 0.02495 | 2 |
| map00500 | Starch and sucrose metabolism | 0.03279 | 0.04743 | 4 |
| map04016 | MAPK signaling pathway | 0.03101 | 0.05618 | 4 |
| map00061 | Fatty acid biosynthesis | 0.05128 | 0.07096 | 2 |
| map00052 | Galactose metabolism | 0.04651 | 0.08409 | 2 |
| map00030 | Pentose phosphate pathway | 0.04545 | 0.08748 | 2 |
| map00051 | Fructose and mannose metabolism | 0.04545 | 0.08748 | 2 |
| map00010 | Glycolysis / Gluconeogenesis | 0.03125 | 0.09304 | 3 |
| map00240 | Pyrimidine metabolism | 0.04255 | 0.09789 | 2 |
| map00710 | Carbon fixation in photosynthetic organisms | 0.03636 | 0.12719 | 2 |
| map04626 | Plant-pathogen interaction | 0.02326 | 0.17720 | 3 |
| map00620 | Pyruvate metabolism | 0.02941 | 0.17840 | 2 |
| map00600 | Sphingolipid metabolism | 0.04762 | 0.21261 | 1 |
| map00909 | Sesquiterpenoid and triterpenoid biosynthesis | 0.04545 | 0.22155 | 1 |
| map00062 | Fatty acid elongation | 0.04348 | 0.23038 | 1 |
| map00460 | Cyanoamino acid metabolism | 0.03226 | 0.29764 | 1 |
| map00640 | Propanoate metabolism | 0.03125 | 0.30563 | 1 |
| map04075 | Plant hormone signal transduction | 0.01527 | 0.34277 | 4 |
| map00900 | Terpenoid backbone biosynthesis | 0.02041 | 0.42864 | 1 |
| map00561 | Glycerolipid metabolism | 0.01852 | 0.46057 | 1 |
| map00630 | Glyoxylate and dicarboxylate metabolism | 0.01754 | 0.47888 | 1 |
| map00260 | Glycine, serine and threonine metabolism | 0.01563 | 0.51928 | 1 |

**Table S10.** Samples of monk fruit and the outgroup *S. siamensis* used in the present study.

| Species | Population | Location | Individuals |
| --- | --- | --- | --- |
| *Siraitia grosvenorii* | Wild population |  |  |
|  | DX | Dao county, Yongzhou city, Hunan province, China | 10 |
|  | GPS | Pinggui, Hezhou city, Guangxi province, China | 10 |
|  | HJ | Huangjiang, Guilin city, Guangxi province, China | 10 |
|  | HP | Wantian, Guilin city, Guangxi province, China | 9 |
|  | JLS | Quannan, Ganzhou city, Jiangxi province, China | 10 |
|  | JPS | Xinfeng, Ganzhou city, Jiangxi province, China | 10 |
|  | JS | Jinshi, Guilin city, Guangxi province, China | 10 |
|  | JX | Jinxiu, Laibin city, Guangxi province, China | 15 |
|  | LS | Lushan, Jiujiang city, Jiangxi province, China | 10 |
|  | NX | Nanxiong, Shaoguan city, Guangdong province, China | 10 |
|  | RY | Ruyuan, Shaoguan city, Guangdong province, China | 6 |
|  | ST | Bobai, Yulin city, Guangxi province, China | 10 |
|  | TD | Bobai, Yulin city, Guangxi province, China | 10 |
|  | WGS | Anfu, Jian city, Jiangxi province, China | 10 |
|  | YF | Yongfu, Guilin city, Guangxi province, China | 9 |
|  |  |  |  |
|  | Cultivated population |  |  |
|  | ZP | Xingan and Yongfu, Guilin city, Guangxi province, China | 18 |
|  |  |  |  |
| *Siraitia siamensis* | Outgroup |  |  |
|  | SS | Yunnan and Guangxi province, China | 6 |

**Table S11.** Genomes of the relevant species used in the present study.

| Species | Accession | Reference |
| --- | --- | --- |
| *Momordica charantia* | PRJNA873243 | Fu *et al*. 2022 |
| *Cucumis sativus* | PRJNA33619 | Li *et al*. 2019 |
| *Citrullus lanatus* | PRJCA008083 | Deng *et al*. 2022 |
| *Cucurbita maxima* | <http://cucurbitgenomics.org/> | Sun *et al*. 2017 |
| *Cucurbita pepo* | PRJNA386743 | Montero-Pau *et al*. 2018 |
| *Cucumis melo* | PRJNA491307 | Zhang *et al*. 2019 |
| *Cucurbita moschata* | <http://cucurbitgenomics.org/> | Sun *et al*. 2017 |
| *Benincasa hispida* | PRJCA022068 | Wang *et al*. 2024 |
| *Arabidopsis thaliana* | <https://www.arabidopsis.org/> | Lamesch *et al*. 2012 |
| *Populus trichocarpa* | PRJNA10772, PRJNA17973 | Tuskan *et al*. 2006 |
| *Vitis vinifera* | <https://grapedia.org/genomes/> | Jaillon *et al*. 2007 |
| *Oryza sativa* | <https://rice.uga.edu/> | Kawahara *et al*. 2013 |
| *Amborella trichopoda* | <https://phytozome-next.jgi.doe.gov/info/Atrichopoda_v1_0> | Amborella Genome Project 2013 |

1. Fu A, Zheng Y, Guo J. *et al*. Telomere-to-telomere genome assembly of bitter melon (*Momordica charantia* L. var. *abbreviata* Ser.) reveals fruit development, composition and ripening genetic characteristics. *Hortic Res*. 2022;10(1):uhac228.
2. Li Q, Li H, Huang W. *et al*. A chromosome-scale genome assembly of cucumber (*Cucumis sativus* L.). *Gigascience*. 2019;8(6):giz072.
3. Deng Y, Liu S, Zhang Y. *et al*. A telomere-to-telomere gap-free reference genome of watermelon and its mutation library provide important resources for gene discovery and breeding. *Mol Plant*. 2022;15(8):1268–84.
4. Sun H, Wu S, Zhang G. *et al*. Karyotype stability and unbiased fractionation in the paleo-allotetraploid *Cucurbita* genomes. *Mol Plant*. 2017;10(10):1293–306.
5. Montero-Pau J, Blanca J, Bombarely A. *et al*. De novo assembly of the zucchini genome reveals a whole-genome duplication associated with the origin of the *Cucurbita* genus. *Plant Biotechnol J*. 2018;16(6):1161–71.
6. Zhang H, Li X, Yu H. *et al*. A high-quality melon genome assembly provides insights into genetic basis of fruit trait improvement. *iScience*. 2019;22:16–27.
7. Wang M, Cao Z, Jiang B. *et al*. Chromosome-level genome assembly and population genomics reveals crucial selection for subgynoecy development in chieh-qua. *Hortic Res*. 2024;11(6):uhae113.
8. Lamesch P, Berardini TZ, Li D. *et al*. The Arabidopsis Information Resource (TAIR): improved gene annotation and new tools. *Nucleic Acids Res*. 2012;40:D1202–10.
9. Tuskan GA, Difazio S, Jansson S. *et al*. The genome of black cottonwood, *Populus trichocarpa* (Torr. & Gray). *Science*. 2006;313(5793):1596–604.
10. Jaillon O, Aury JM, Noel B. *et al*. The grapevine genome sequence suggests ancestral hexaploidization in major angiosperm phyla. *Nature*. 2007;449(7161):463–7.
11. Kawahara Y, de la Bastide M, Hamilton JP. *et al*. Improvement of the *Oryza sativa* Nipponbare reference genome using next generation sequence and optical map data. *Rice*. 2013;6(1):4.
12. Amborella Genome Project. The *Amborella* genome and the evolution of flowering plants. *Science*. 2013;342(6165):1241089.
